# Incidence of Immune Checkpoint Inhibitor-Associated Diabetes: A Meta-Analysis of Randomized Controlled Studies

**DOI:** 10.3389/fphar.2019.01453

**Published:** 2019-12-06

**Authors:** Jingli Lu, Jing Yang, Yan Liang, Haiyang Meng, Junjie Zhao, Xiaojian Zhang

**Affiliations:** ^1^Department of Pharmacy, the First Affiliated Hospital of Zhengzhou University, Zhengzhou, China; ^2^Henan Key Laboratory of Precision Clinical Pharmacy, Zhengzhou University, Zhengzhou, China

**Keywords:** immune checkpoint inhibitors, diabetes, hyperglycemia, meta-analysis, safety outcomes

## Abstract

**Background:** Immune checkpoint inhibitors (ICIs) are now an important option for more than 14 different cancers. Recent series case reports have described that ICIs are associated with new-onset diabetes in patients, yet the definitive risk is not available. We thus performed a meta-analysis of randomized controlled trials (RCTs) to assess the incidence and risk of developing new-onset diabetes following the use of ICIs.

**Methods:** The PubMed, EMBASE, Cochrane Library databases, and ClinicalTrials.gov for RCTs were searched. Statistical analyses were performed using STATA 15 and R language. Fifty-two RCTs were included, and 12 did not report any events of ICI-associated diabetes.

**Results:** A meta-analysis of 40 trials was performed, which reported at least one diabetes-related event among 24,596 patients. Although specific diabetes-related events were rare, compared with the placebo or other therapeutic strategies, the rates of serious hyperglycemia (OR 2.41, 95% CI 1.52 to 3.82), diabetes (3.54, 1.32 to 9.51), all-grade T1D (6.60, 2.51 to 17.30), and serious-grade T1D (6.50, 2.32 to 18.17) were increased with ICI drugs. Subgroup analysis according to the type of control, type of ICIs, and the combination mode suggested that ICIs plus conventional treatments significantly decreased the risks of diabetes and serious-grade hyperglycemia. There was little heterogeneity across the studies in all results except hyperglycemic events, which in part was attributable to data from everolimus-based control group.

**Conclusions:** New-onset diabetes is uncommon with ICIs but the risk is increased compared with placebo or another therapeutic strategy. However, more studies are warranted to substantiate these findings across ICIs.

## Introduction

Immune checkpoint inhibitor (ICI)-based treatments that block molecules such as programmed cell death protein 1 (PD-1), PD1 ligand 1 (PD-L1), and cytotoxic T lymphocyte-associated antigen 4 (CTLA-4) have emerged as powerful weapons in a growing number of cancers (Temel et al., 2018). Currently, nine ICIs have been approved for the treatment of different cancers: anti-PD-1 (nivolumab, pembrolizumab, toripalimab, sintilimab, and cemiplimab); anti-PD-L1 (atezolizumab, avelumab, and durvalumab); and anti-CTLA-4 (ipilimumab). Immune checkpoint molecules play an important role in maintaining immunological tolerance to self-antigens and preventing autoimmune disorders (Pardoll, 2012). Consequently, their blockade in cancer therapy not only promotes T cell-mediated immune destruction on tumor cells but may also facilitate autoimmune activity that affects various organ systems (Johnson et al., 2018). Thus, ICIs frequently cause toxicities related to the mechanism of action that are generally referred to as immune-related adverse events (irAEs) (Postow et al., 2018).

Among these irAEs, new-onset diabetes is receiving increased attention, as more evidence suggests the recognition of diabetes-related adverse events in patients with cancers who are treated with ICIs. A marked increase in reporting diabetes has also been seen since 2017 by analyzing the World Health Organization’s database of individual case safety reports (Wright et al., 2018). These observations raised concern as to whether ICI treatments could be associated with an increased risk of diabetes in patients with cancer. However, there has been no report of a meta-analysis of the incidence or risk of ICI-associated diabetes among the different ICIs in different tumor subtypes.

Given the dramatic growth in the number of clinical trials testing ICI agents and their clinical benefits in the increasing list of cancer types and negative influence on life quality caused by diabetes if not promptly recognized, we performed a meta-analysis of randomized controlled trials (RCTs) with ICIs in patients with cancer and evaluated the incidence and risks of diabetes-related adverse events compared with placebo or another therapeutic strategy.

## Methods

### Search Strategy and Selection Criteria

Scientific literature searches were performed in three databases (PubMed, EMBASE, and Cochrane Central Register of Controlled Trials) from the inception of all searched databases to March 2019. Relevant text words and medical subject headings that consisted of terms including ‘phase’ and the individual drug names (details in Supporting Information **Table S1**) were searched. The search was limited to RCTs and English language. We also performed a manual search using reference lists from trials and review articles to identify any other relevant data. The ClinicalTrials.gov website was searched for RCTs that were labeled as ‘completed’ with available results. This meta-analysis was performed in adherence with the Preferred Reporting Items for Systematic Reviews and Meta-Analyses guidelines (Moher et al., 2009).

### Study Selection

We included RCTs that were performed in adults with cancer and compared ICI treatment to another treatment strategy. The exclusion criteria were as follows: observational and retrospective studies; studies published in a meeting abstract without published full text original articles; quality of life studies; studies with only pediatric patients; 10 or fewer patients in any group; single dosing; cost effectiveness analyses; and those that could not assess the effect of ICI, such as when the control group was a different dose of the same ICI or another type of ICI. Two authors independently screened all titles and abstracts (HM and JZ). Two of three authors reviewed and discussed the potential full text. Any disagreements were resolved by consensus with all three (JL, HM, and JZ).

### Data Extraction and Quality Assessment

Data from each study that met the inclusion criteria were independently extracted by two of the three authors (JL, HM, and YL). Any disagreement was resolved by consensus with all three. The retrieved data included author name, year of publication, trial characteristics (registry number, whether it was an international study, countries involved, study sites, and study phase), patient characteristics (sex, age, and performance status), the sizes of the intervention and control groups, ICI treatment, dose, and the outcomes of interest. We detected new-onset diabetes following treatment with ICIs using the following terms: hyperglycemia, diabetes mellitus (DM), type 2 diabetes (T2D), and type 1 diabetes (T1D). For data extracted from ClinicalTrials.gov, adverse events were reported as either serious or other; for data from published reports, we identified grades 3–5 as serious and grades 1–2 as other, according to Common Terminology of Clinical Adverse Events categorization. If data were available for both sources, we prioritized data from sources where the data were more complete. If a published study did not report diabetes-related adverse events, and the corresponding registry trial from ClinicalTrials.gov reported did, we included the registry report. For multiple reports of the same trial, only the most completely reported data were used. The quality of the included studies was independently assessed using the Cochrane Risk of Bias Tool. We considered all trials at unclear risk of incomplete outcome data and selective reporting bias as these studies were not designed primarily to assess adverse events.

### Data Synthesis and Analysis

The estimated event rates in the intervention group are calculated as the total number of patients with a given adverse event divided by the total number at risk. Data were transformed using the Freeman-Tukey Double Arcsine transformation to calculate event rates. This statistical analyses were performed using R statistical software (package meta, R Foundation). For risk outcome, we pooled trials and calculated odds ratios (ORs) and their associated 95% confidence intervals (CIs) in the intervention group compared with the control group based on the number of patients with a given adverse event and sample size. Given the low rates of adverse events, we used Peto’s method to pool effect estimates across studies. The *I*² statistic and *P* value were used to examine heterogeneity across trials for each outcome. An *I*² statistic of 0–25%, 26–75%, and 76–100% was regarded as indicating low, moderate, and high heterogeneity, respectively. A *P* value of less than or equal to 0.05 was defined as significant heterogeneity. If a study included more than one intervention group (e.g. different doses or different types of ICI), we separately compared each intervention group with the control group, where the number of patients or events in the control group would be doubled. Sensitivity analyses were performed excluding an everolimus-controlled study, which was known to cause diabetes-related adverse events, to understand the reasons for the high likelihood of differences. We conducted subgroup analyses to examine studies according to the type of control group (chemotherapy vs. immunosuppressive drug vs. targeted therapy vs. placebo), the mode of intervention treatment (monotherapy vs. add-on therapy), and the type of ICI (PD-1 vs. PD-L1 vs. CTLA4 vs. combination of ICIs). Evidence of publication bias was assessed using Egger’s and Begg’s test in addition to funnel plots, and significant publication bias defined as a *P* < 0.1. All statistical analyses were conducted with STATA, version 15.

## Results

### Study Search

**Figure 1:**
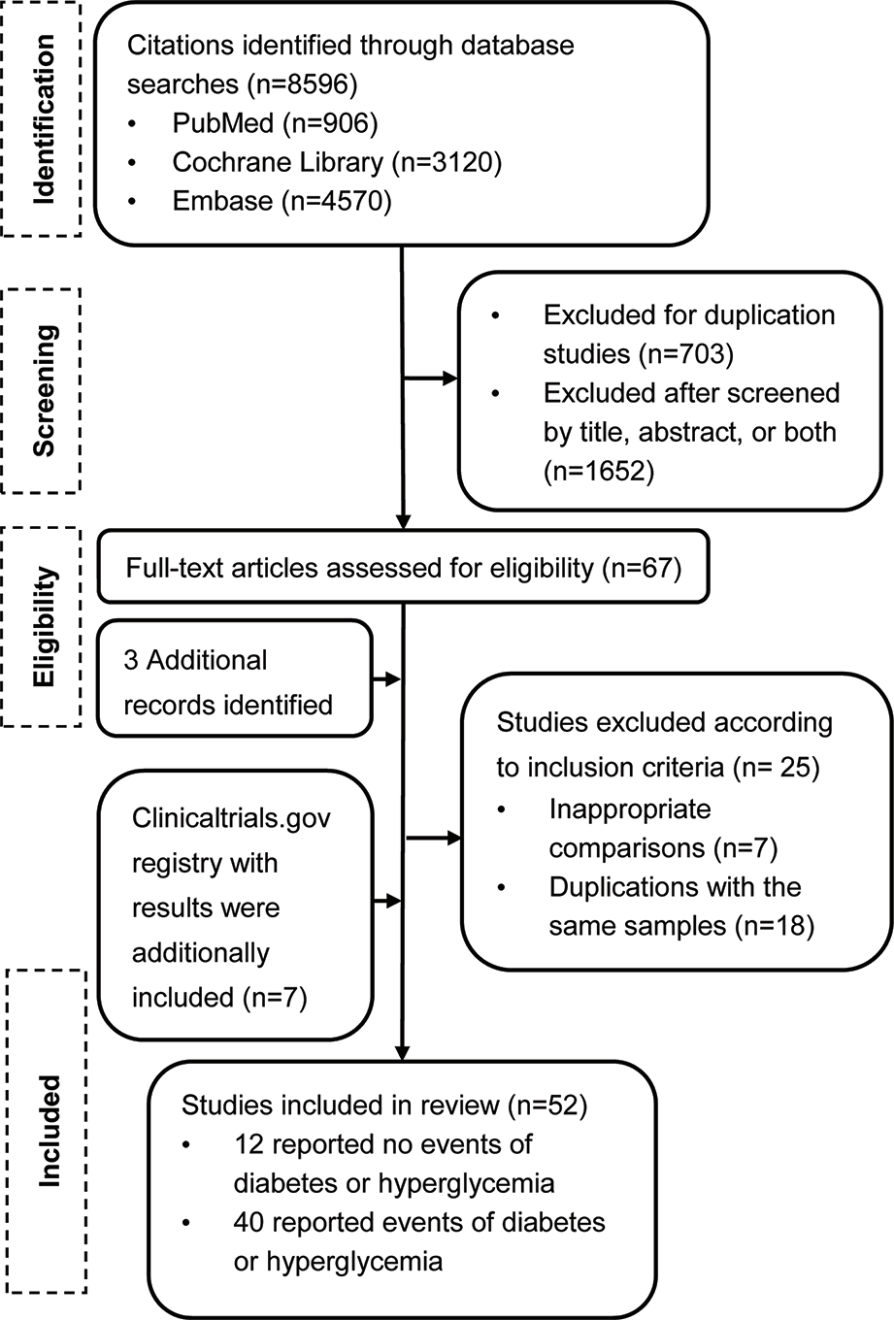
Flow diagram of study selection.

Our search from the PubMed, EMBASE, and Cochrane Central Register databases yielded a total of 8,596 potentially relevant reports (**Figure 1**). After screening and eligibility assessment, we retrieved 67 reports for full text screening. We also identified 117 reports with results from ClinicalTrial.gov. After our formal search, three additional large clinical trials were published. We therefore also included these three studies. After further section, a total of 52 studies (7 from the trial registry and 45 from journals) were eligible. The included articles were published (online) between August 2010 and April 2019.

### Study Characteristics

All studies except one (Chih-Hsin Yang et al., 2019) were international multicenter studies. All studies were funded by the pharmaceutical industry, with sample sizes of the ICI intervention group ranging from 12 to 636 patients. Twenty-two were completed in patients with non-small-cell lung cancer, eight in melanoma, six in renal cell carcinoma, three in small-cell lung cancer, three in gastric and gastro esophageal junction cancer, two in head and neck squamous cell carcinoma, two in urothelial cancer, two in prostate cancer, two in breast cancer, one in colorectal cancer, and one in mesothelioma. Among these, patients in the intervention arm received nivolumab as monotherapy in ten studies, pembrolizumab in seven studies, atezolizumab in five studies, durvalumab in three studies, avelumab in one study, tremelimumab in three studies, combination therapy with anti-PD-1/PD-L1/CTLA-4 plus chemotherapy/radiotherapy in thirteen studies, combination therapy with anti-PD-1/PD-L1 plus anti-CTLA4 in three studies, combination therapy with anti-PD-1/PD-L1/CTLA-4 plus targeted therapy in seven studies, and combination therapy with ipilimumab plus vaccine in one study. All studies except one (Kang et al., 2017) had adverse event data on ClinicalTrials.gov. Key characteristics of these included trials are shown in **Table 1**.

**Table 1:** Characteristics of controlled trials of ICI treatment in patients.

NCT Author (year)	International study	No. of countries involved	No. of study sites	Phase	Group type	Drug	Dose of ICI (mg/kg)	No. of patients	Age Median (range)	No (%) Male	Tumor type
NCT00527735 (Reck et al., 2013)	Yes	8	NR	Phase 2	CTLA4	Ipilimumab Paclitaxel/carboplatin	10	113	NR	NR	NSCLC
					CTLA4	Ipilimumab Paclitaxel/carboplatin	10	109	NR	NR	
					Control	Paclitaxel/carboplatin	/	109	NR	NR	
NCT00861614 (Kwon et al., 2014)	Yes	26	191	Phase 3	CTLA4	Ipilimumab Radiotherapy	10	399	69 (47–86)	399	Prostate cancer
					Control	Placebo radiotherapy	/	400	67.5 (45–86)	400	
NCT01673867 (Borghaei et al., 2015)	Yes	22	NR	Phase 3	PD-1	Nivolumab	3	292	61 (37–84)	151 (52)	NSCLC
					Control	Docetaxel	/	290	64 (21–85)	168 (58)	
NCT01642004 (Brahmer et al., 2015)	Yes	20	NR	Phase 3	PD-1	Nivolumab	3	135	62 (39–85)	111 (82)	NSCLC
					Control	Docetaxel	/	137	64 (42–84)	97 (71)	
NCT00636168 (Eggermont et al., 2015)	Yes	19	91	Phase 3	CTLA4	Ipilimumab	10	475	51 (20–84)	296 (62)	Melanoma
					Placebo	Placebo	/	476	52 (18–78)	293 (62)	
NCT01668784 (Motzer et al., 2015)	Yes	24	146	Phase 3	PD-1	Nivolumab	3	410	62 (23–88)	315 (77)	RCC
					Control	Everolimus	/	411	62 (18–86)	304 (74)	
NCT01704287 (Ribas et al., 2015)	Yes	12	73	Phase 2	PD-1	Pembrolizumab	2	180	62 (15–87)	104 (58)	Melanoma
					PD-1	Pembrolizumab	10	181	60 (27–89)	109 (60)	
					Control	Carboplatin/paclitaxel Dacarbazine Temozolomide	/	179	63 (27–87)	114 (64)	
NCT01721772 (Robert et al., 2015)	Yes	16	80	Phase 3	PD-1	Nivolumab	3	210	64 (18–86)	121 (57.6)	Melanoma
					Control	Dacarbazine	/	208	66 (26–87)	125 (60.1)	
NCT01721746 (Weber et al., 2015)	Yes	14	90	Phase 3	PD-1	Nivolumab	3	272	59 (23–88)	176 (65)	Melanoma
					Control	Dacarbazine/carboplatin/paclitaxel	/	133	62 (29–85)	85 (64)	
NCT01903993 (Fehrenbacher et al., 2016)	Yes	13	61	Phase 2	PD-L1	Atezolizumab	1,200 mg/dose	144	62 (42–82)	93 (65)	NSCLC
					Control	docetaxel	/	143	62 (36–84)	76 (53)	
NCT02105636 (Ferris et al., 2016)	Yes	15	NR	Phase 3	PD-1	Nivolumab	3	240	59 (29–83)	197 (82.1)	HNSCC
					Control	Cetuximab/methotrexate/docetaxel	/	121	61 (28–78)	103 (85.1)	
NCT01905657 (Herbst et al., 2016)	Yes	24	202	Phase 2/3	PD-1	Pembrolizumab	2	344	63 (56–69)	212 (62)	NSCLC
					PD-1	Pembrolizumab	10	346	63 (56–69)	213 (62)	
					Control	Docetaxel	/	343	62 (56–69)	209 (61)	
NCT02039674 (Langer et al., 2016)	Yes	2	26	Phase 2	PD-1	Pembrolizumab Carboplatin/pemetrexed	200 mg/dose	60	62.5 (54–70)	22 (37)	NSCLC
					Control	Carboplatin/pemetrexed	/	63	63.2 (58–70)	26 (41)	
NCT01450761 (Reck et al., 2016a)	Yes	34	224	Phase 3	CTLA4	Ipilimumab Etoposide/cisplatin/carboplatin	10	478	62 (39–85)	371 (66)	SCLC
					Control	Placebo Etoposide/cisplatin/carboplatin	/	476	63 (36–81)	326 (68)	
NCT02142738 (Reck et al., 2016b)	Yes	16	142	Phase 3	PD-1	Pembrolizumab	200 mg/dose	154	64.5 (33–90)	92 (59.7)	NSCLC
					Control	Paclitaxel/carboplatin/pemetrexed/cisplatin/gemcitabine	/	151	66 (38–85)	95 (62.9)	
NCT02008227 (Rittmeyer et al., 2017)	Yes	31	194	Phase 3	PD-L1	Atezolizumab	1,200 mg/dose	613	NR	378 (61.7)	NSCLC
					Control	Docetaxel	/	612	NR	379 (61.9)
NCT02125461 (Antonia et al.,2017)	Yes	26	235	Phase 3	PD-L1	Durvalumab	10	476	64 (31–84)	334 (70.2)	NSCLC
					Control	Placebo	/	237	64 (23–90)	166 (70)	
NCT01057810 (Beer et al., 2017)	Yes	24	NR	Phase 3	CTLA4	Ipilimumab	10	399	NR	100	Prostate cancer
					Control	Placebo	/	199	NR	100	
NCT02256436 (Rogers et al., 2017)	Yes	120	29	Phase 3	PD-1	Pembrolizumab	200 mg/dose	270	67 (29–88)	200 (74.1)	Urothelial carcinoma

					Control	Paclitaxel/docetaxel/vinflunine	/	272	65 (26–84)	202 (74.3)
NCT02041533 (Carbone et al., 2017)	Yes	26	NR	Phase 3	PD-1	Nivolumab	3	271	63 (32–89)	184 (68)	NSCLC
					Control	Gemcitabine/cisplatin Carboplatin/paclitaxel/pemetrexed					

							/	270	65 (29–87)	148 (55)	
NCT01285609 (Govindan et al., 2017)	Yes	34	233	Phase 3	CTLA4	Ipilimumab Paclitaxel/carboplatin	10	479	NR	NR	NSCLC
					Control	Placebo Paclitaxel/carboplatin	/	477	NR	NR	
NCT02267343 (Kang et al., 2017)	Yes	3	49	Phase 3	PD-1	Nivolumab	3	330	62 (54–69)	229 (69)	GEJ
					Control	Placebo	/	163	61 (53–68)	119 (73)	
NCT01843374 (Llombart-Cussac et al., 2017)	Yes	19	105	Phase 2b	CTLA4	Tremelimumab	10	382	66 (60–72)	283 (74.1)	Mesothelioma
					Control	Placebo	/	189	67 (61–73)	151 (79.9)	
NCT02302807 (Powles et al., 2018)	Yes	29	217	Phase 3	PD-L1	Atezolizumab	1,200 mg/dose	467	67 (33–88)	357 (76)	Urothelial bladder
					Control	Vinflunine/paclitaxel/docetaxel	/	464	67 (31–84)	361 (78)	cancer
NCT02395172 (Barlesi et al., 2018)	Yes	31	173	Phase 3	PD-L1	Avelumab	10	396	64 (58–69)	269 (67.9)	NSCLC
					Control	Docetaxel	/	396	63 (57–69)	273 (68.9)	
NCT02362594 (Eggermont et al., 2018)	Yes	23	123	Phase 3	PD-1	Pembrolizumab	200 mg/dose	514	54 (19–88)	324 (63)	Melanoma
					Control	Placebo	/	505	54 (19–83)	304 (60.2)	
NCT02578680 (Gandhi et al., 2018)	Yes	16	126	Phase 3	PD-1	Pembrolizumab Pemetrexed/cisplatin	200 mg/dose	410	65 (34–84)	254 (62)	SCLC
					Control	Pemetrexed/cisplatin	/	206	63.5 (34–84)	109 (52.9)	
NCT02231749 (Motzer et al., 2018)	Yes	28	175	Phase 3	PD-1/CTLA4	Nivolumab ipilimumab	3 1	550	NR	NR	RCC
					Control	sunitinib	/	546	NR	NR	
NCT02775435 (Paz-Ares et al., 2018)	Yes	17	137	Phase 3	PD-1	Pembrolizumab Paclitaxel/nab-paclitaxel/carboplatin	200 mg/dose	278	65 (29–87)	220 (79.1)	NSCLC
					Control	Paclitaxel/Nab-paclitaxel/Carboplatin	/	281	65 (36–88)	235 (83.6)	
NCT02370498 (Shitara et al., 2018)	Yes	30	148	Phase 3	PD-1	Pembrolizumab	200 mg/dose	296	62.5 (54–70)	202 (68)	GEJ
					Control	Pacitraxel	/	296	60.0 (53–68)	208 (70)	
NCT02252042 (Cohen et al., 2019)	Yes	20	97	Phase 3	PD-1	Pembrolizumab	200 mg/dose	247	60.0 (55–66)	207 (84)	HNSCC
					Control	Methotrexate Docetaxel/cetuximab	/	248	60.0 (54–66)	205 (83)	
NCT02788279 (Eng et al., 2019)	Yes	11	73	Phase 3	PD-L1	Atezolizumab Cobimetinib	840 mg/dose	183	58 (51–67)	107 (58)	Colorectal cancer
					PD-L1	Atezolizumab	1,200 mg/dose	90	56 (51–64)	59 (66)	
					Control	Regorafenib	/	90	59 (52–66)	51 (57)	
NCT02220894 (Mok et al., 2019)	Yes	32	213	Phase 3	PD-1	Pembrolizumab	200 mg/dose	636	63 (56–69)	450 (71)	NSCLC
					Control	Platinum	/	615	63 (57–69)	452 (71)	
NCT02613507 (Wu et al., 2019)	Yes	3	32	Phase 3	PD-1	Nivolumab	3	338	60 (27–78)	236 (78)	NSCLC
					Control	Docetaxel	/	166	60 (38–78)	134 (81)	
NCT02454933 (Chih-Hsin Yang et al., 2019)	No	1	1	Phase 3	PD-L1	Durvalumab Osimertinib	10 mg/kg	12	56 (41–78)	6 (50)	NSCLC
					Control	Osimertinib	/	17	65 (41–80)	4 (24)	
NCT01585987 (Squibb, 2012)	Yes	12	NR	Phase 2	CTLA4	Ipilimumab	10 mg/kg	57	NR	NR	GEJ
					Control	Fluoropyrimidine	/	57	NR	NR	
NCT01984242 (Roche, 2014)	Yes	9	NR	Phase 2	PD-L1	Atezolizumab Bevacizumab	1,200 mg/dose	101	NR	74 (73.3)	RCC
					PD-L1	Atezolizumab	1,200 mg/dose	103	NR	77 (74.8)
					Control	Sunitinib	/	101	NR	79 (78.2)
NCT02367781 (Roche, 2015b)	Yes	36	NR	Phase 3	PD-L1	Atezolizumab Nab-paclitaxel/carboplatin	1,200 mg/dose	483	NR	NR	NSCLC
					Control	Nab-paclitaxel/carboplatin	/	240	NR	NR
NCT02352948 (AstraZeneca, 2015)	Yes	NR	82	Phase 3 subA	PD-L1	Durvalumab	10	62	NR	42 (67.7)	NSCLC
					PD-L1/CTLA4	Durvalumab Tremelimumab	20 1	174	NR	115 (66.1)	
	Yes	NR	143	Phase 3 subB	Control	Eerlotinib/gemcitabine/vinorelbine	/	64	NR	48 (75.0)	
					PD-L1	Durvalumab	10	117	NR	73 (62.4)	
					CTLA4	Tremelimumab	10	60	NR	39 (65.0)	
					Control	Gemcitabine/vinorelbine	/	118	NR	81 (68.6)	
NCT02420821 (Roche, 2015a)	Yes	21	NR	Phase 3	PD-L1	Atezolizumab Bevacizumab	1,200 mg/dose	451	NR	NR	RCC
					Control	Sunitinib	/	446	NR	NR
NCT00094653 (Hodi et al., 2010)	Yes	13	125	Phase 3	CTLA4	Ipilimumab gp100	3	403	55.6^a^	247 (61.3)	Melanoma
					CTLA4	Ipilimumab	3	137	56.8^a^	81 (59.1)	
					Control	gp100	/	136	57.4^a^	73 (53.7)	
NCT00324155 (Robert et al., 2011)	Yes	NR	25	Phase 3	CTLA4	Ipilimumab Dacarbazine	10	250	57.5^a^	152 (60.8)	Melanoma
					Control	Dacarbazine	/	252	56.4^a^	149 (59.1)	
(Lynch et al., 2012)	Yes	NR	NR	Phase 2	CTLA4	Ipilimumab Paclitaxel/carboplatin	10	70	NR	NR	NSCLC
					CTLA4	Ipilimumab Paclitaxel/carboplatin	10	68	NR	NR	
					Control	Paclitaxel/carboplatin	/	66	NR	NR	
NCT00257205 (Ribas et al., 2013)	Yes	24	114	Phase 3	CTLA4	Tremelimumab	15	328	57^a^	190 (58)	Melanoma
					Control	Dacarbazine/temozolomide	/	327	56^a^	182 (56)	
NCT02477826 (Hellmann et al., 2017)	Yes	36	NR	Phase 3	PD-1/ CTLA4	Nivolumab/ipilimumab	3 1	396	NR	NR	PD-L1 expression≥1% NSCLC
					PD-1	Nivolumab	240 mg/dose	396	NR	NR	
					Control	Platinum	/	397	NR	NR	
					PD-1/CTLA4	Nivolumab/ipilimumab	3 1	187	NR	NR	PD-L1 expression
					PD-1	Nivolumab	360 mg/dose	177	NR	NR	<1% NSCLC
					Control	Platinum	/	186	NR	NR	
NCT02763579 (Horn et al., 2018)	Yes	21	106	Phase 3	PD-L1	Atezolizumab Carboplatin/etoposide	1,200 mg/dose	201	64 (28-90)	129 (64.2)	SCLC
					Control	Carboplatin/etoposide	/	202	64 (26-87)	132 (65.3)
NCT02425891 (Schmid et al., 2018)	Yes	41	246	Phase 3	PD-L1	Atezolizumab Nab-paclitaxel	840 mg/dose	451	55 (20-82)	3 (0.7)	Breast cancer
					Control	Placebo nab-paclitaxel	/	451	56 (26-86)	1 (0.2)	
NCT02366143 (Socinski et al., 2018)	Yes	26	240	Phase 3	PD-L1	Atezolizumab Bevacizumab/barboplatin/paclitaxel	1,200 mg/dose	400	63 (31-89)	240 (60.0)	NSCLC
					PD-L1	Atezolizumab Carboplatin/paclitaxel	1,200 mg/dose	402	NR	NR	
					Control	Bevacizumab/carboplatin/paclitaxel	/	400	63 (31-90)	239 (59.8)	
NCT02684006 (Motzer et al., 2019)	Yes	21	144	Phase 3	PD-L1	Avelumab Axitinib	10	442	62 (29-83)	316 (71.5)	RCC
					Control	Sunitinib	/	444	61 (27-88)	344 (77.5)	
NCT02853331 (Rini et al., 2019)	Yes	16	129	Phase 3	PD-1	Pembrolizumab Axitinib	200 mg/dose	432	62 (30-89)	308 (71.3)	RCC
					Control	Sunitinib	/	429	61 (26-90)	320 (74.6)	
NCT02250326 (Celgene, 2015)	Yes	7	34	Phase 2	PD-L1	Nab-Paclitaxel Durvalumab	1,125 mg/m^2^	79	NR	54 (68.4)	NSCLC
					Control	Nab-paclitaxel	/	80	NR	50 (62.5)	
					Control	Nab-paclitaxel CC-486	/	81	NR	50 (61.7)	
NCT02924883 (Roche, 2016)	Yes	9		Phase 3	PD-L1	Atezolizumab Trastuzumab/emtansine	1,200 mg/dose	133	NR	2 (1.5)	Breast cancer
					Control	Placebo Trastuzumab/emtansine	/	69	NR	0 (0.0)	

GEJ, gastric and gastroesophageal junction cancer; HNSCC, head and neck squamous cell carcinoma; ICI, immune checkpoint inhibitors; NR, not reported; NSCLC, non-small-cell lung cancer; RCC, renal cell carcinoma; SCLC, small-cell lung cancer. ^a^Age mean.

### Quality of the Included Studies

**Table S2** shows the risk of bias assessment of the included studies for meta-analysis. All studies were RCTs with adequate reported randomization, and all studies were funded by the pharmaceutical industry with a high risk of sponsorship bias. Of the 40 included studies for meta-analysis, 26 (65%) were open labels with a high risk of blinding participants and personnel. None of the included studies specifically stated blinded assessment or collection of diabetes-related adverse events. We classified all trials at unclear risk of incomplete outcome data and selective reporting bias.

### Incidence of Diabetes-Related Adverse Events

Of the 52 clinical controlled trials assessing the effects of ICIs, 40 trials described ICI-associated diabetes events during the course of study. Hyperglycemia events were described in 32 studies; 303 cases of all-grade hyperglycemia and 55 serious-grade hyperglycemia events occurred in 10,393 patients. Pooling the data showed that the rates of all-grade and serious-grade hyperglycemia events were 2.26% (95% CI, 1.28 to 3.48) and 0.28% (95% CI, 0.16 to 0.42), respectively. The rates of hyperglycemia events differed by the type of ICI and tumor. In particular, patients treated with ICI combination therapy were more likely to report hyperglycemia: 3.37% for all-grade hyperglycemia events, 0.47% for serious-grade hyperglycemia. Patients with RCC showed a trend toward higher rates of both all-grade and serious-grade hyperglycemia, with rates of 6.82% and 0.66%, respectively. High dose of ICIs was not associated with high rates of hyperglycemia events (**Table 2**).

Due to the smaller number of other ICI-associated diabetes events, no statistical inferences of the rates were made. Overall, 13 cases of DM occurred in 5,655 patients (raw event rate 0.23%), five cases of T2D occurred in 3,117 patients (raw event rate 0.16%), and 17 cases of all-grade T1D occurred in 3,899 patients (raw event rate 0.44%), and 15 cases of serious-grade T1D events occurred in 3,603 patients (raw event rate 0.42%).

**Table 2:** Incidence of hyperglycemia events in patients treated with immune checkpoint inhibitors. Values are percentages (95% confidence intervals).

Characteristic	All-grade hyperglycemia	Serious-grade hyperglycemia
**Total**	2.26 (1.28, 3.48)	0.28 (0.16, 0.42)
**ICI type**		
PD-1 inhibitors	4.86 (2.86, 7.32)	0.49 (0.26, 0.78)
PD-L1 inhibitors	0.81 (0.07, 2.06)	\
CTLA-4 inhibitors	0.52 (0.09, 1.18)	0.06 (0.00, 0.28)
Combination therapy	3.37 (0.00, 21.49)	0.47 (0.00, 2.01)
**Tumor type**		
NSCLC	2.54 (1.10, 4.43)	0.22 (0.06, 0.45)
Melanoma	1.75 (0.31, 4.15)	0.35 (0.09, 0.73)
RCC	6.82 (2.00, 14.05)	0.66 (0.27, 1.18)
Prostate cancer	0.12^a^	0.12^a^
Colorectal cancer	0.37^a^	/
GEJ	0.57^a^	0.53^a^
HNSCC	5.42^a^	0.42^a^
Mesothelioma	0.52^a^	0.52^a^
SCLC	0.63^a^	0.63^a^
**Dose**		
High dose	1.33 (0.27, 2.99)	0.22 (0.00, 0.80)
Normal dose	2.52 (1.32, 4.03)	0.28 (0.15, 0.44)

GEJ, gastric and gastroesophageal junction cancer; HNSCC, head and neck squamous cell carcinoma; NSCLC, non-small-cell lung cancer; RCC, renal cell carcinoma; SCLC, small-cell lung cancer.

High doses: including Ipilimumab 10 mg/kg and pembrolizumab 10 mg/kg.

a Raw event rate.

### Risk of Diabetes-Related Adverse Events

**Figure 2:**
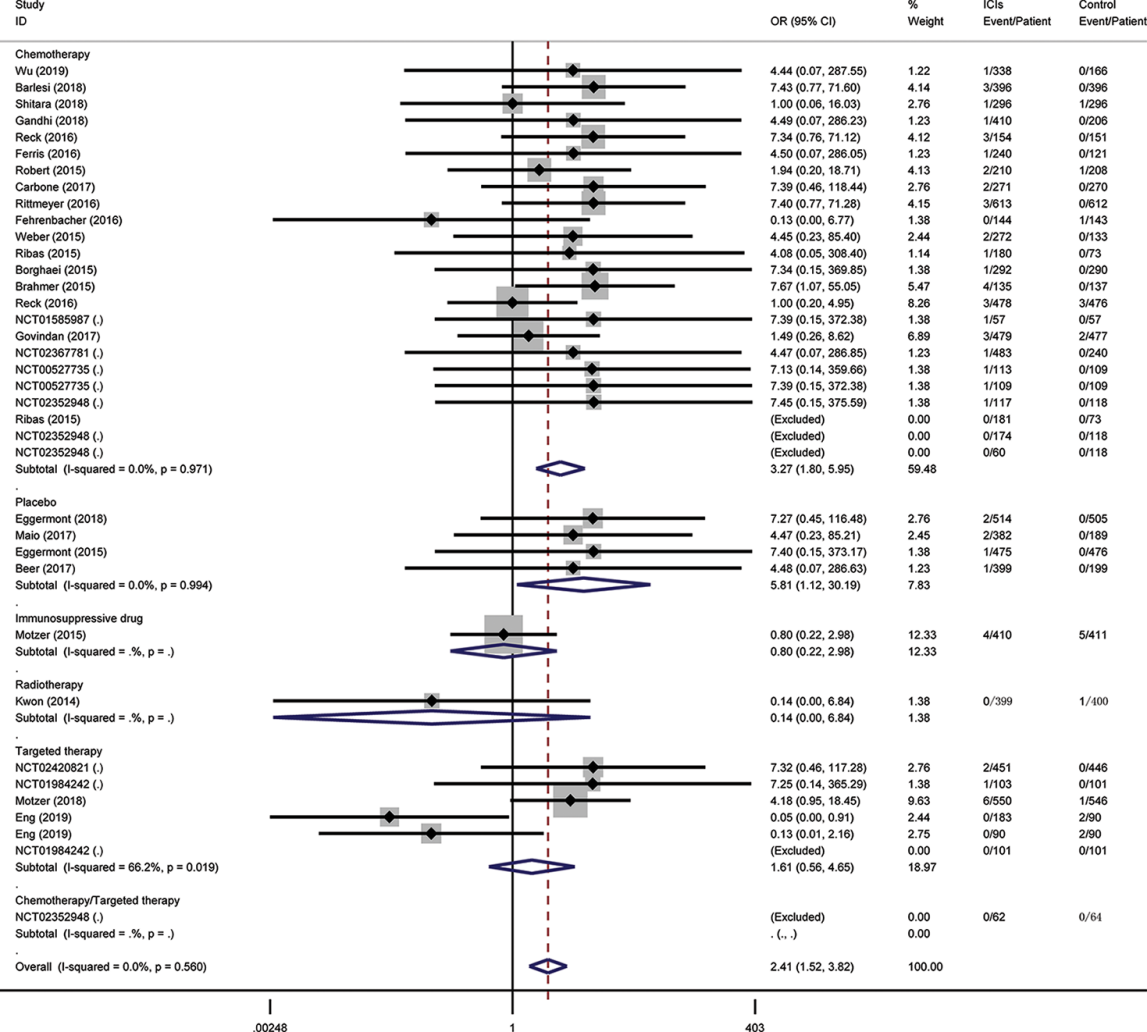
Risk of serious-grade hyperglycemia following the use of ICIs versus control treatment, stratified by the type of control group.

**Figure 3:**
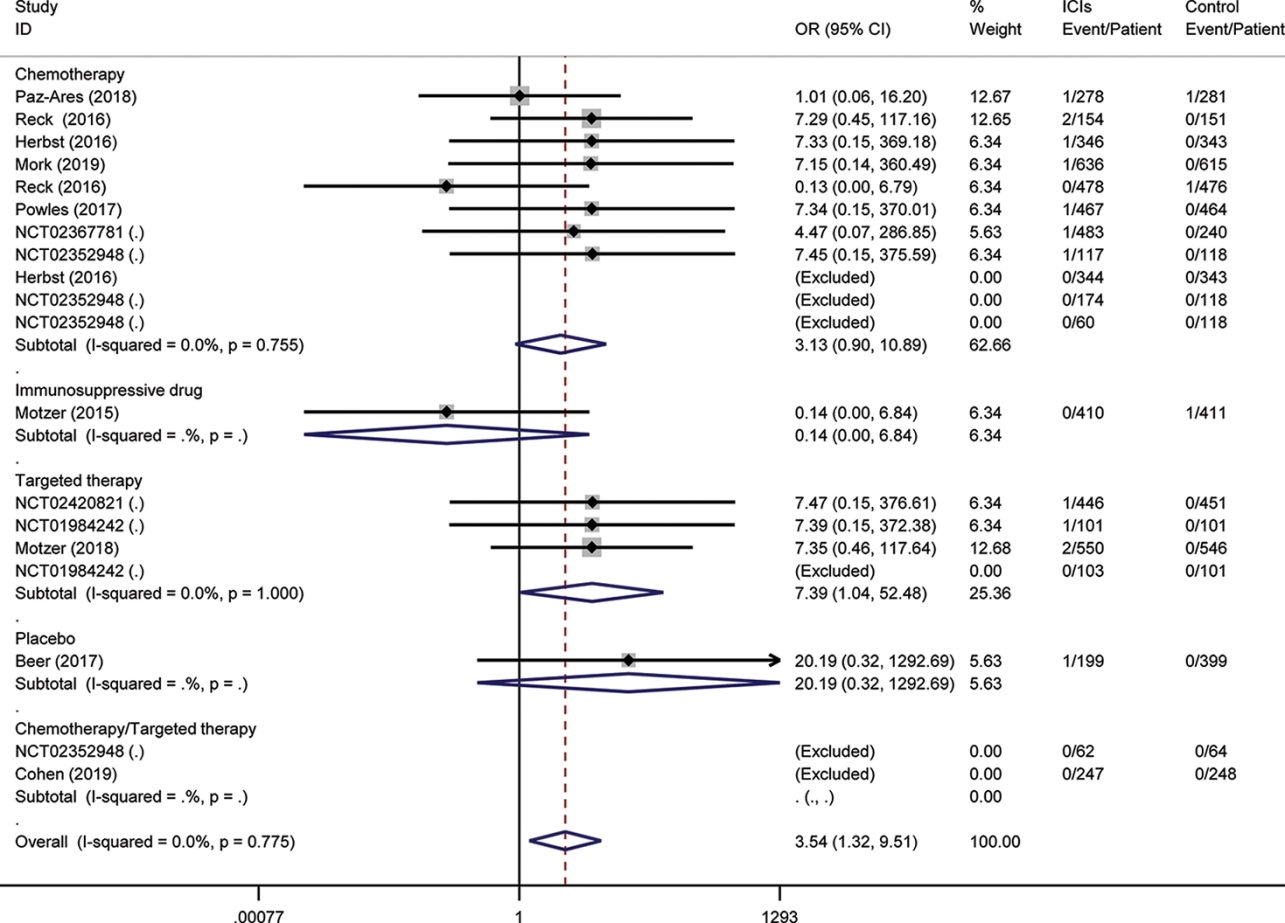
Risk of diabetes mellitus following the use of ICIs versus control treatment, stratified by the type of control group.

**Figure 4:**
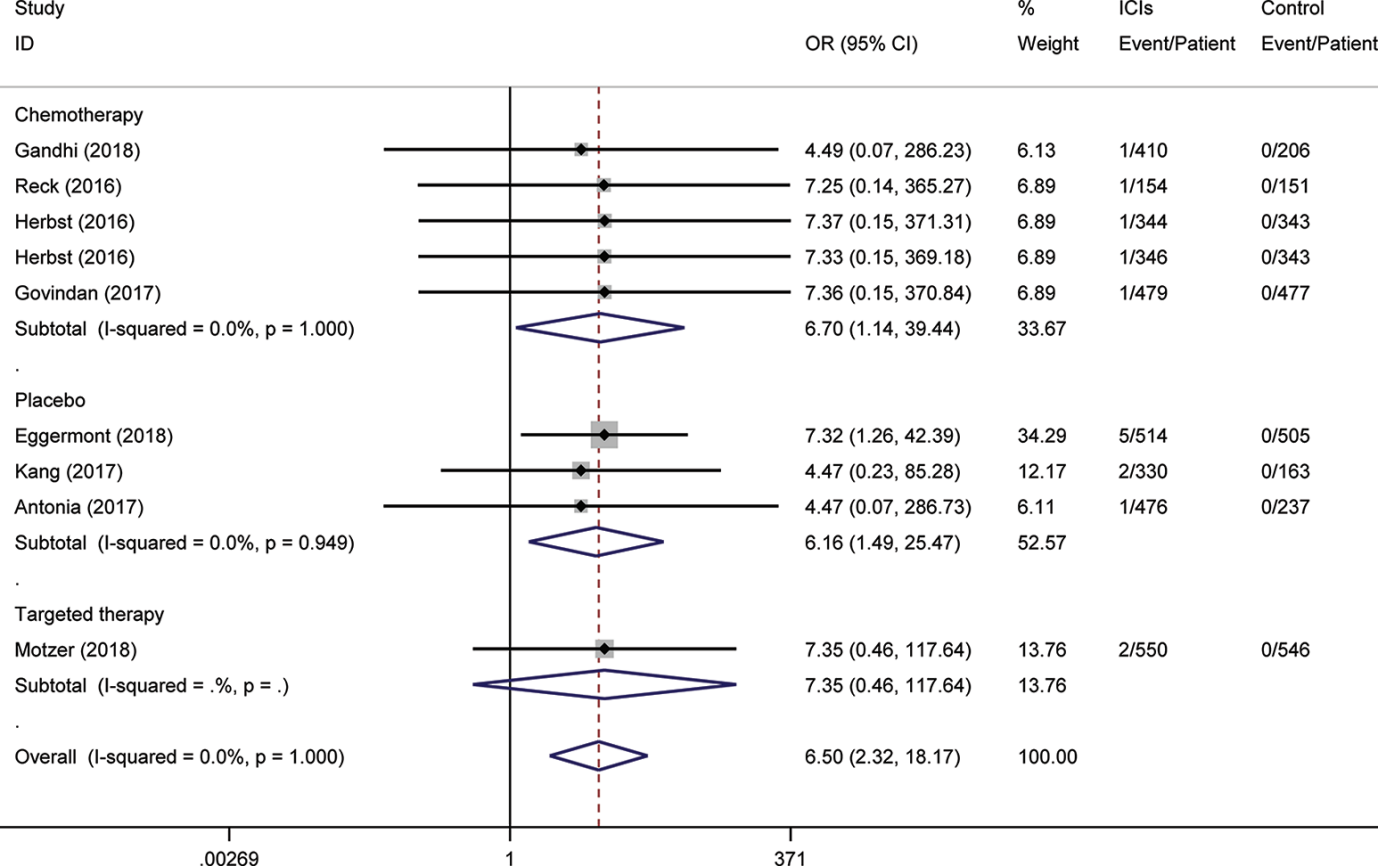
Risk of serious-grade type 1 diabetes following the use of ICIs versus control treatment, stratified by the type of control group.

To assess the relative rate of ICI-associated diabetes compared with those in control arms, we calculated the OR of developing diabetes in the RCTs. Pooling the data of these studies showed that patients treated with ICIs were at higher risk for serious-grade hyperglycemia (OR 2.41, 95% CI 1.52 to 3.82, **Figure 2**), DM (OR 3.54, 95% CI 1.32 to 9.51, **Figure 3**), all-grade T1D (OR 6.60, 95% CI 2.51 to 17.30, **Figure S1**), and serious-grade T1D (OR 6.50, 95% CI 2.32 to 18.17, **Figure 4**) than those treated with other regimens. ICIs showed a trend toward an increased risk of all-grade hyperglycemia (OR 1.38, 95% CI 1.15 to 1.66, **Figure S2**), but no increased risk of T2D (OR 0.92, 95% CI 0.24 to 3.52, **Figure S3**). Excluding the study in which the control group was everolimus, a drug known to cause diabetes, the risk of ICI-associated diabetes events were also higher than the control: OR 4.42 for DM, OR 1.75 for all-grade hyperglycemia, OR 2.81 for serious-grade hyperglycemia (**Figures S4–S6**).

Subgroup analysis for these outcomes was stratification by the type of control, the mode of treatment, and type of ICI. Regarding the type of control, there were apparent differences across subgroups for the risk of ICI-associated diabetes events. Within the placebo-controlled group, ICIs were associated with a higher risk in hyperglycemia (OR 5.81). Subgroup analysis based on the mode of treatment (monotherapy vs. add-on therapy) suggests that add-on therapy decreased the risk of ICI-associated diabetes, with OR 1.77 for DM, 1.31 for serious-grade hyperglycemia, 0.58 for T2D, and 5.83 for T1D (**Figures S7–S11**). The subgroup analysis by the type of ICI suggests the risk of these events was increased in the subset of trials in which anti-PD-1 or anti-PD-L1 was combined with anti-CTLA-4, with OR 7.35 for DM, 2.51 for all-grade hyperglycemia, 4.18 for serious-grade hyperglycemia (**Figures S12–S17**).

The funnel plot and statistical test showed no evidence of publication bias for DM (Egger’s test *P* = 0.994), all-grade hyperglycemia (Egger’s test *P* = 0.128), serious-grade hyperglycemia (Egger’s test *P* = 0.325), T2D (Egger’s test *P* = 0.310), all-grade T1D (Egger’s test *P* = 0.300), and serious-grade T1D (Egger’s test *P* = 0.334) (**Table S3**, **Figures S18–S23**). We noted no heterogeneity in the effects of ICI on DM, serious-grade hyperglycemia, T2D, all-grade T1D, and serious-grade T1D (*I*² = 0.0%). However, we noted substantial heterogeneity for the outcome of all-grade hyperglycemia (*I*² = 88.2%), which was considerably reduced in the analyses of data excluding the everolimus-controlled study (*I*² = 8.0%).

## Discussion

We completed a systematic analysis of new-onset diabetes following treatment with ICIs versus other therapeutic regimens to further our understanding of the safety of these agents. We used data from 40 RCTs that included 13,787 patients treated with ICIs, and also extracted data from the ClinicalTrials.gov results database to supplement the published studies. To our knowledge, this is the largest and most comprehensive meta-analysis on the incidence and risk of ICI-associated diabetes events following the use of ICI regimens published to date, although previous case series analyses showed that there is an increased reporting of rapidly progressive ICI-associated diabetes (Wright et al., 2018; Kotwal et al., 2019; Perdigoto et al., 2019). This meta-analysis shows that the risk of serious-grade hyperglycemia, DM, and T1D following ICIs is significantly higher compared with patients treated with other regimens, but provides no support that ICI treatment is associated with an increased risk of all-grade of hyperglycemia. Among patients on each different ICI regimens, patients on combination therapy were more likely to develop hyperglycemia.

Although the incidence was low, T1D has emerged as the highest risk associated with ICI therapy compared with other diabetes-related adverse events. The pathogenesis of T1D in the populations of patients receiving ICIs is not currently well understood. Several case reports have shown that the presence of autoantibodies before ICIs-based therapy might be at risk of developing diabetes, particularly in treated with anti-PD-1/anti-PD-L1 (Gauci et al., 2017; Usui et al., 2017; Way et al., 2017). Further support for autoimmune-based mechanism has been shown by Clotman et al. (2018), who overviewed the reported cases and demonstrated that approximately half of the tested cases of ICI-associated T1D had detectable diabetes-related autoantibodies. Other studies have shown that anti-PD-1 resulted in a rapid progression of autoimmune diabetes in patients with a high underlying genetic predisposition to T1D (Mellati et al., 2015), raising the concern for genetic factors as a possible mechanism in patients with diabetes-prone HLA genotypes. Similar to what has been described in humans, the study demonstrated that PD-1 or PD-L1 blockade rapidly precipitated diabetes in prediabetic nonobese diabetic (NOD) mice (Ansari et al., 2003). Taken together, these studies reveal a potential mechanism of ICI-associated T1D that involves in both diabetes-related immunologic and genetic factors.

The subgroup analysis showed that the risk of ICI-associated T1D was different among the different type of ICIs. One possible explanation for this would be the mechanistic link to each target. Unlike the PD-1 pathway, which modulates effector cells, CTLA-4 functions in early immune responses during T cell priming and activation (Topalian et al., 2016). As such, the distinct function of the PD-1 and CTLA4 potentially contributed to different rates of T1D following the use of ICIs. In NOD mice, CTLA-4 blockade negatively physiologically regulated diabetes in only the early stages of life compared with the PD-1 pathway (Ansari et al., 2003). Additionally, there was strong PD-L1 expression in the inflamed islets of NOD mice, which suggested that the PD-1-mediated regulation of autoreactive immune cells played an important role at the site of islet inflammation (Ansari et al., 2003). However, this finding should be interpreted cautiously; more data are needed for definitive conclusions given the low absolute number of T1D in patients receiving ICIs.

ICIs plus conventional treatments have been tested in multiple solid tumors, which achieved synergetic effects and overcame the resistance to immunotherapy (Yan et al., 2018). When we combined all non-ICI therapy into one control category, the ICI-based regimens substantially increased the risk of ICI-associated diabetes compared with control group. However, this magnitude was reduced when ICIs were used as an add-on therapy. The risk of DM was 200% lower in the add-on therapy than in the ICI monotherapy. There was also a substantial reduction (over 175%) in ICI-associated serious-grade hyperglycemia in the setting of conventional treatments. These results consistently suggested that compared with ICI therapy, ICIs plus traditional therapy could result in a decreased risk of diabetes-related adverse events.

We found little heterogeneity across studies for all results except hyperglycemia, which strengthens the primary conclusion that ICIs increased risks of diabetes events. A sensitivity analysis identified that everolimus-based control group is responsible for this heterogeneity. Everolimus is an mTOR inhibitor, which is known to influence insulin signaling pathway in peripheral tissues and insulin secretion in *pancreatic* β *cells* (Tuo and Xiang, 2018). It has described that mTOR inhibitors resulted in a 5-fold increase in the risk for severe hyperglycemia in patients with cancer (Verges, 2018). Thus, when everolimus was presented separately, the heterogeneity was reduced.

There are several limitations in the present study. We conducted this analysis in study-level, rather than individual patient data. It is not possible to assess potential risk factors that are associated with higher risk of new-onset diabetes, due to the lack of detailed clinical data such as sex, diabetes-prone HLA genotypes, presence of autoantibodies, and islet function in patients receiving ICIs therapy. Secondly, subgroup effects could not be evaluated when there were less than two trials in each subgroup, which could not allow assessing whether the rates of ICI-associated diabetes are varied based on the type of tumor and the dose of ICIs. Our results showed that high dose of ICIs did not contribute to high rates of hyperglycemia events, while the type of tumor showed association of treatment effects. However, regarding other diabetes symptoms, we pooled data across studies together, which might result in the missed difference in dose-dependent and tumor-dependent effect on the risk for these adverse events. Thirdly, whether the increased risk of hyperglycemic events were caused, at least partly, by the use of corticosteroids for the management of irAEs is unclear. Moreover, the results of the present analysis are unable to address potential associations between the incidence of new-onset diabetes and other irAEs in the individual-level. Lastly, only very recent publications have noted T1D after ICI therapy; our study therefore may have underestimated the prevalence of ICI-associated diabetes with only a focus on clinical trials. As emerging case reports that described new-onset diabetes were seen in clinical practice (Hughes et al., 2015; Martin-Liberal et al., 2015; Wright et al., 2018), these adverse events may become more accurately diagnosed and recorded in future trials.

In summary, the use of ICIs compared with placebo or other treatment strategies was associated with an increased risk of new-onset diabetes, especially autoimmune diabetes, although the overall event rates remained low. In contrast, compared with the control group, the risk of T2D was not increased. As the widespread awareness of these events increases, additional large, well-designed randomized trials are needed to definitively determine the risks of new-onset diabetes following the use of ICIs.

## Data Availability Statement

The datasets generated for this study are available on request to the corresponding author.

## Author Contributions

JL, JY, and XZ conceived and designed the study. YL, HM, JZ, JL, and JY reviewed the literatures, extracted and analyzed the data. JL, JY, and XZ wrote the manuscript. All authors have read and approved the final manuscript.

## Funding

This work was supported by the National Natural Science Foundation of China (grant number 81603122 to JL).

## Conflict of Interest

The authors declare that the research was conducted in the absence of any commercial or financial relationships that could be construed as a potential conflict of interest.
