# Supplementary material for: Incidence of Immune Checkpoint Inhibitor-Associated Diabetes: A Meta-Analysis of Randomized Controlled Studies
Source: Front Pharmacol. 2019 Dec 6;10:1453. doi: 10.3389/fphar.2019.01453 (PMC6915045; doi:10.3389/fphar.2019.01453)
Supplement: Supplementary file 1 [file DataSheet_1.pdf]

Supporting information

Supplementary Table S1. Search strategy.

Data source	Search terms
**PubMed**	ALL FIELDS (ipilimumab OR MDX-010 OR tremelimumab OR CP-675,206 OR nivolumab OR BMS-963558 OR pembrolizumab OR MK-3475 OR atezolizumab OR MPDL3280A OR avelumab OR MSB0010718C OR durvalumab OR MEDI4736 OR Cemiplimab OR REGN2810 OR Toripalimab OR JS001 OR Sintilimab OR IBI308) AND (phase)
**Cochrane library**	ALL TEXT ipilimumab OR MDX-010 OR tremelimumab OR CP-675,206 OR nivolumab OR BMS-963558 OR pembrolizumab OR MK-3475 OR atezolizumab OR MPDL3280A OR avelumab OR MSB0010718C OR durvalumab OR MEDI4736 OR Cemiplimab OR REGN2810 OR Toripalimab OR JS001 OR Sintilimab OR IBI308
**Embase**	ALL FIELDS ('phase '/exp OR 'phase') AND ('ipilimumab'/exp OR 'ipilimumab' OR 'MDX-010'/exp OR 'MDX-010' OR 'tremelimumab'/exp OR 'tremelimumab' OR 'CP-675,206 '/exp OR 'CP-675,206' OR 'nivolumab '/exp OR 'nivolumab' OR 'BMS-963558'/exp OR 'BMS-963558' OR 'pembrolizumab'/exp OR 'pembrolizumab' OR 'MK-3475'/exp OR 'MK-3475' OR 'atezolizumab'/exp OR 'atezolizumab' OR 'MPDL3280A'/exp OR 'MPDL3280A' OR 'avelumab'/exp OR 'avelumab' OR 'MSB0010718C'/exp OR 'MSB0010718C' OR 'pembrolizumab'/exp OR 'pembrolizumab' OR 'MK-3475'/exp OR 'MK-3475' OR 'atezolizumab'/exp OR 'atezolizumab' OR 'MPDL3280A'/exp OR 'MPDL3280A' OR 'avelumab'/exp OR 'avelumab' OR 'MSB0010718C'/exp OR 'MSB0010718C' OR 'durvalumab'/exp OR 'durvalumab' OR 'MEDI4736'/exp OR 'MEDI4736' OR 'Cemiplimab'/exp OR 'Cemiplimab' OR 'REGN2810'/exp OR 'REGN2810' OR 'Toripalimab'/exp OR 'Toripalimab' OR 'JS001'/exp OR 'JS001' OR 'Sintilimab'/exp OR 'Sintilimab' OR 'IBI308'/exp OR 'IBI308')
**ClinicalTrials.gov**	(ipilimumab OR MDX-010 OR tremelimumab OR CP-675,206 OR nivolumab OR BMS-963558 OR pembrolizumab OR MK-3475 OR atezolizumab OR MPDL3280A OR avelumab OR MSB0010718C OR durvalumab OR MEDI4736 OR Cemiplimab OR REGN2810 OR Toripalimab OR JS001 OR Sintilimab OR IBI308)

Supplementary Table S2. Risk of bias of clinical trials for meta-analysis

No.	Study	Random sequence generation	Allocation concealment	Blinding of participants and personnel	Blinding of outcome assessment	Incomplete outcome data	Selective reporting	Sponsorship bias	Other bias
1	Recks, 2013(Reck et al., 2013) NCT00527735	Low	Low	Low	Low	Unclear	Unclear	High	Unclear
2	Kwon, 2014(Kwon et al., 2014) NCT00861614	Low	Low	Low	Low	Unclear	Unclear	High	Unclear
3	Borghaei, 2015(Borghaei et al., 2015) NCT01673867	Low	Unclear	High	Unclear	Unclear	Unclear	High	Unclear
4	Brahmer, 2015(Brahmer et al., 2015) NCT01642004	Low	Unclear	High	Unclear	Unclear	Unclear	High	Unclear
5	Eggermont, 2015(Eggermont et al., 2015) NCT00636168	Low	Low	Low	Low	Unclear	Unclear	High	Unclear
6	Motzer, 2015(Motzer et al., 2015)	Low	Unclear	High	Unclear	Unclear	Unclear	High	Unclear

7	NCT01668784 Ribas, 2015(Ribas et al., 2015)	Low	Low	Low	Low	Unclear	Unclear	High	Unclear
8	NCT01704287 Robert, 2015(Robert et al., 2015)	Low	Low	Low	Low	Unclear	Unclear	High	Unclear
9	NCT01721772 Weber, 2015(Weber et al., 2015)	Low	Unclear	High	Low	Unclear	Unclear	High	Unclear
10	NCT01721746 Fehrenbacher, 2016 (Fehrenbacher et al., 2016)	Low	High	High	Unclear	Unclear	Unclear	High	Low
11	NCT01903993 Ferris, 2016 (Ferris et al., 2016)	Low	Unclear	High	High	Unclear	Unclear	High	Unclear
12	NCT02105636 Herbst, 2016 (Herbst et al., 2016)	Low	Unclear	High	High	Unclear	Unclear	High	Unclear
13	NCT01905657 Langer, 2016(Langer et al.,	Low	Unclear	High	High	Unclear	Unclear	High	Unclear

	2016)								
14	NCT02039674 Reck, 2016(Reck et al., 2016a)	Low	Low	Low	Low	Unclear	Unclear	High	Unclear
15	NCT01450761 Reck, 2016(Reck et al., 2016b)	Low	Unclear	High	Unclear	Unclear	Unclear	High	Unclear
16	NCT02142738 Rittmeyer, 2016(Rittmeyer et al., 2017)	Low	High	High	Unclear	Unclear	Unclear	High	Unclear
17	NCT02008227 Antonia, 2017(Antonia et al., 2017)	Low	Low	Low	Low	Unclear	Unclear	High	Unclear
18	NCT02125461 Beer, 2017(Beer et al., 2017)	Low	Low	Low	Low	Unclear	Unclear	High	Unclear
19	NCT01057810 Bellmunt, 2017(Rogers et al., 2017)	Low	Unclear	High	High	Unclear	Unclear	High	Unclear
20	NCT02256436 Carbone, 2017(Carbone et al., 2017)	Low	Unclear	High	High	Unclear	Unclear	High	Unclear

21	NCT02041533 Govindan, 2017(Govindan et al., 2017)	Low	Low	Low	Low	Unclear	Unclear	High	Unclear
22	NCT01285609 Kang, 2017(Kang et al., 2017)	Low	Low	Low	Low	Unclear	Unclear	High	Unclear
23	NCT02267343 Maio, 2017(Llombart-Cussac et al., 2017)	Low	Low	Low	Low	Unclear	Unclear	High	Unclear
24	NCT01843374 Powles, 2017 (Powles et al., 2018)	Low	Unclear	High	High	Unclear	Unclear	High	Unclear
25	NCT02302807 Barlesi, 2018(Barlesi et al., 2018)	Low	Unclear	High	High	Unclear	Unclear	High	Unclear
26	NCT02395172 Eggermont, 2018(Eggermont et al., 2018)	Low	Low	Low	Low	Unclear	Unclear	High	Unclear
27	NCT02362594 Gandhi, 2018(Gandhi et	Low	Low	Low	Low	Unclear	Unclear	High	Unclear

	al., 2018)								
28	NCT02578680 Motzer, 2018 (Motzer et al., 2018)	Low	Unclear	High	High	Unclear	Unclear	High	Unclear
29	NCT02231749 Paz-Ares, 2018(Paz-Ares et al., 2018)	Low	Low	Low	Low	Unclear	Unclear	High	Unclear
30	NCT02775435 Shitara, 2018(Shitara et al., 2018)	Low	Unclear	High	Low	Unclear	Unclear	High	Unclear
31	NCT02370498 Cohen, 2019(Cohen et al., 2019)	Low	Unclear	High	Unclear	Unclear	Unclear	High	Unclear
32	NCT02252042 Eng, 2019(Eng et al., 2019)	Low	Unclear	High	High	Unclear	Unclear	High	Unclear
33	NCT02788279 Mork, 2019(Mok et al., 2019)	Low	Unclear	High	High	Low	Unclear	High	Unclear
34	NCT02220894 Wu, 2019(Wu et al., 2019)	Low	Unclear	High	High	Unclear	Unclear	High	Unclear

35	NCT02613507 Yang, 2019(Chih-Hsin Yang et al., 2019)	Low	Unclear	High	High	Unclear	Unclear	High	Unclear
36	NCT02454933 NCT01585987(Squibb, 2012)	Low	Unclear	High	High	Unclear	Unclear	High	Unclear
37	NCT01984242(Roche, 2014)	Low	Unclear	High	High	Unclear	Unclear	High	Unclear
38	NCT02367781(Roche, 2015b)	Low	Unclear	High	High	Unclear	Unclear	High	Unclear
39	NCT02352948(AstraZeneca, 2015)	Low	Unclear	High	High	Unclear	Unclear	High	Unclear
40	NCT02420821(Roche, 2015a)	Low	Unclear	High	High	Unclear	Unclear	High	Unclear

Supplementary Table S3 The results of publication bias test based on the Egger's test and Begg's test

**Outcomes**	**Egger's test**	**Begg's test**
DM	0.994	0.855
All-grade hyperglycemia	0.128	0.733
Serious-grade hyperglycemia	0.325	0.092
T2D	0.310	0.063
All-grade T1D	0.300	0.004
Serious-grade T1D	0.334	0.016

Abbreviations: DM, diabetes mellitus; T1D, type 1 diabetes; T2D, type 2 diabetes.

Supplementary Figure S1. Risk of all-grade T1D following the use of ICIs versus control treatment, stratified by the type of control group.

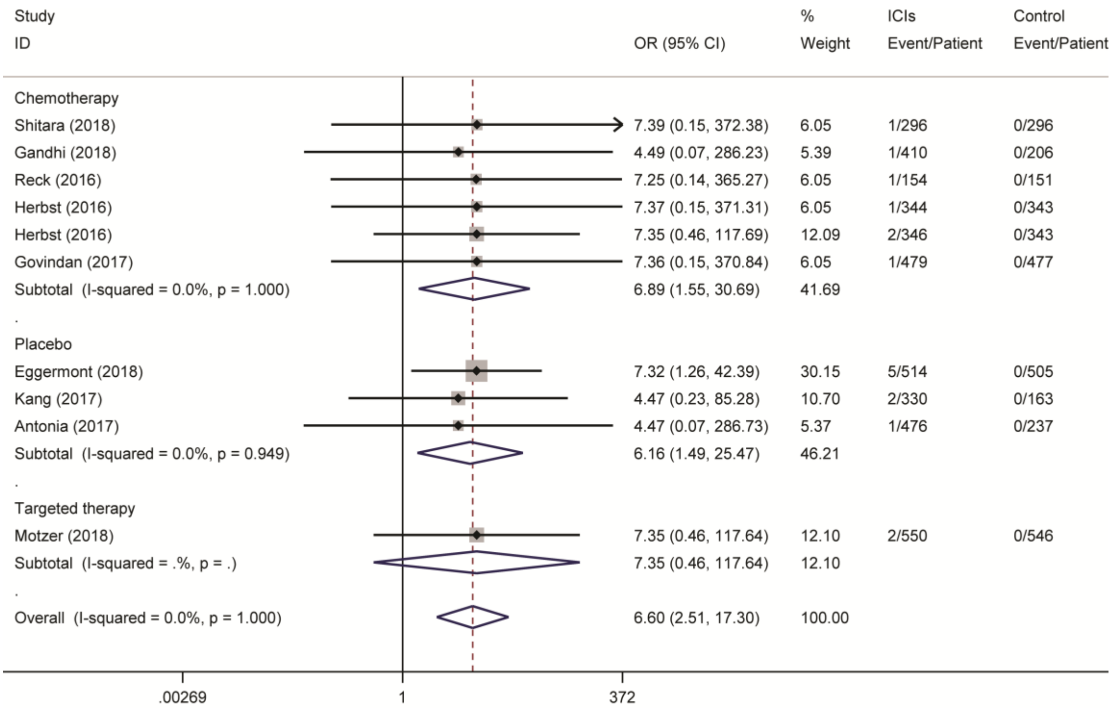

Supplementary Figure S2. Risk of all-grade hyperglycemia following the use of ICIs versus control treatment, stratified by the type of control group.

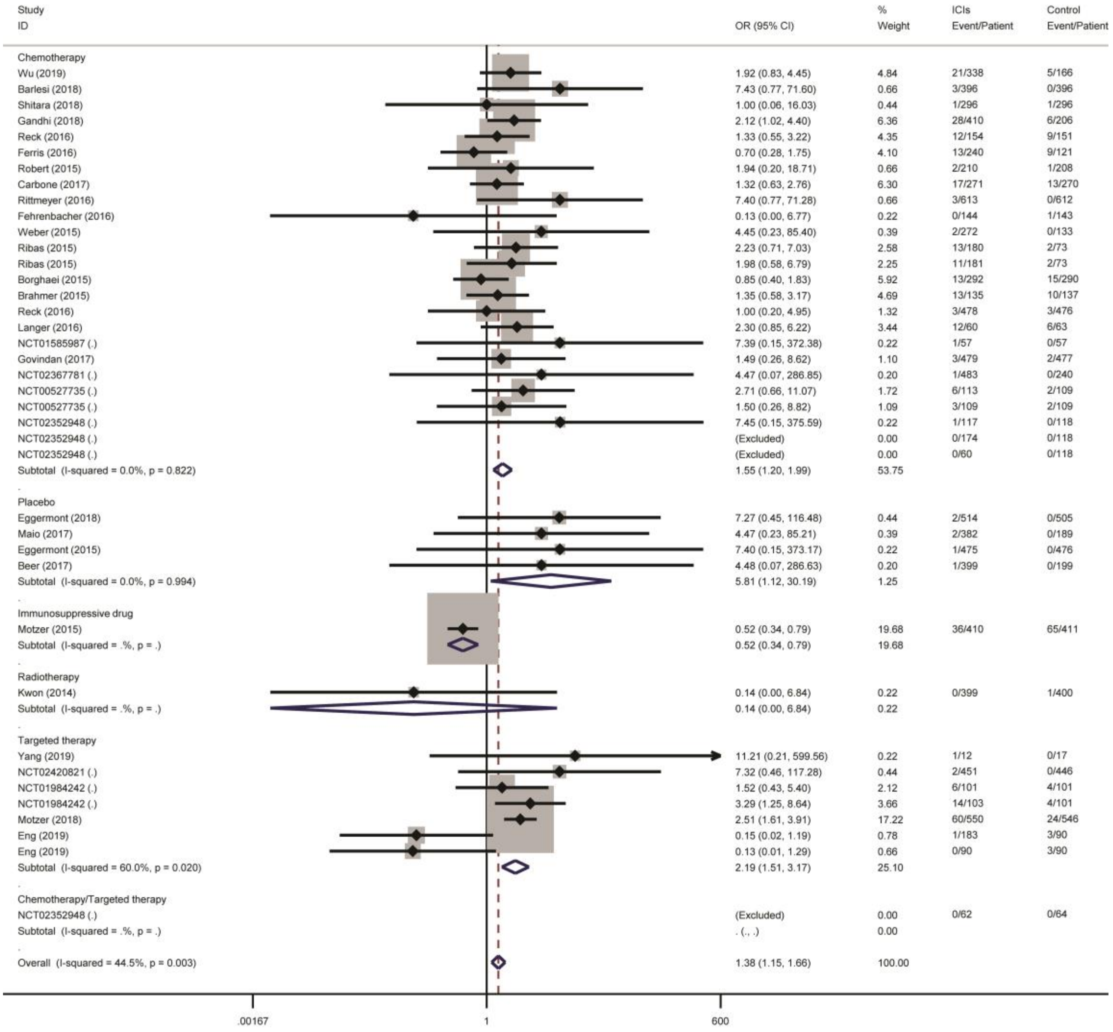

Supplementary Figure S3. Risk of type 2 diabetes following the use ICIs of versus control treatment, stratified by the type of control group.

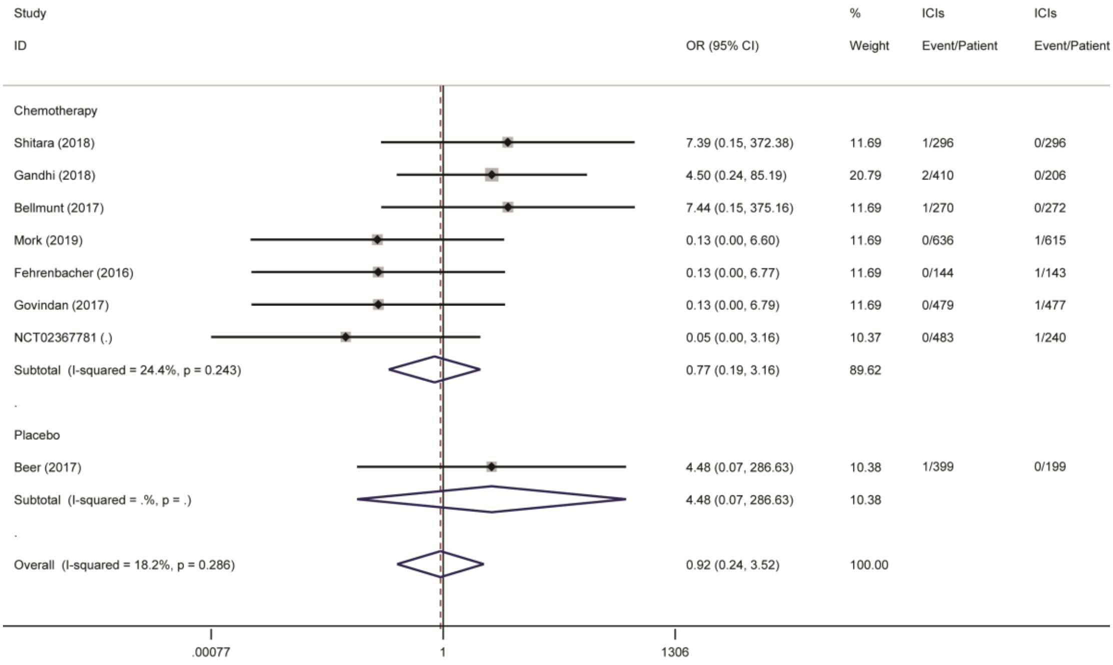

Supplementary Figure S4. Risk of diabetes mellitus following the use of ICIs versus control treatment, after excluding everolimus-based control group.

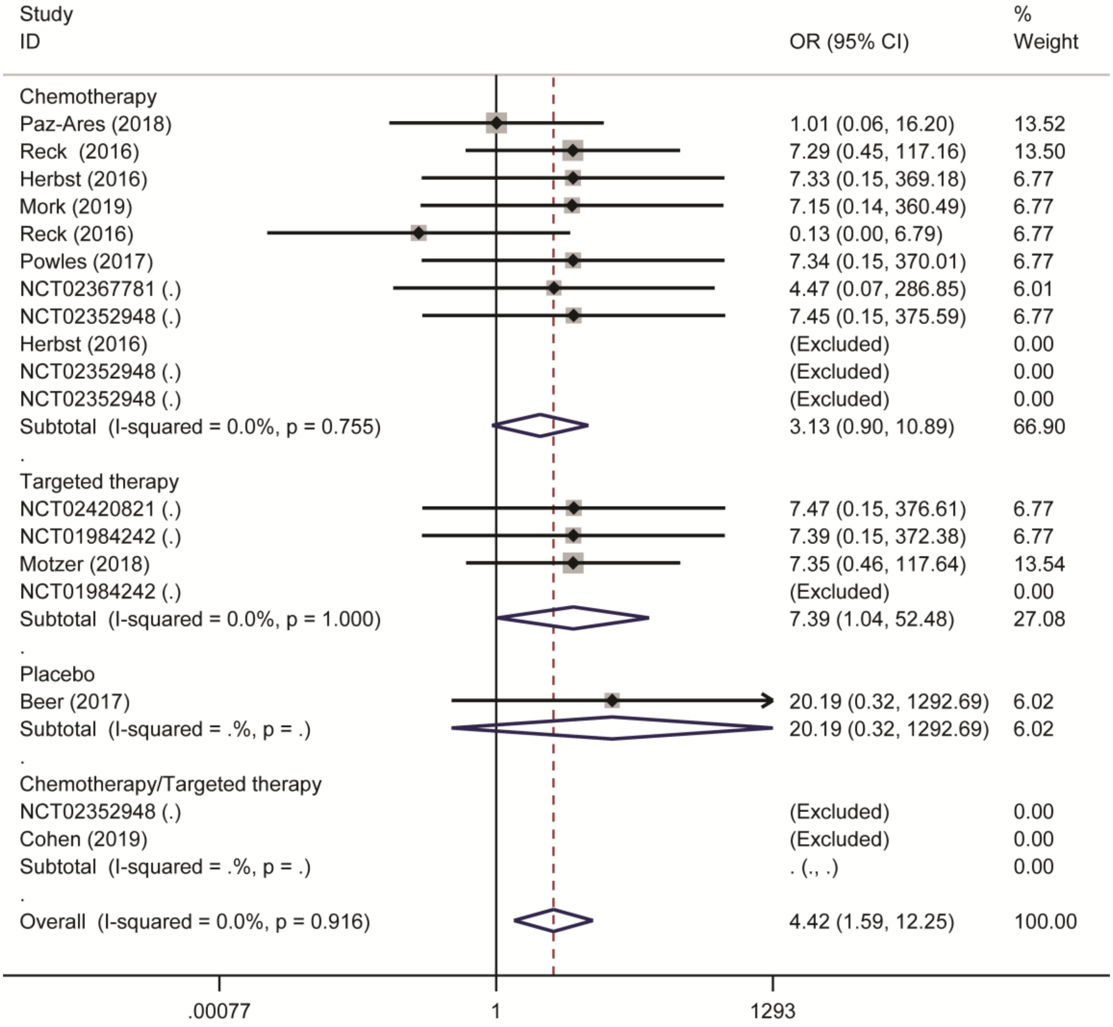

Supplementary Figure S5. Risk of all-grade hyperglycemia following the use of ICIs versus control treatment, after excluding everolimus-based control group.

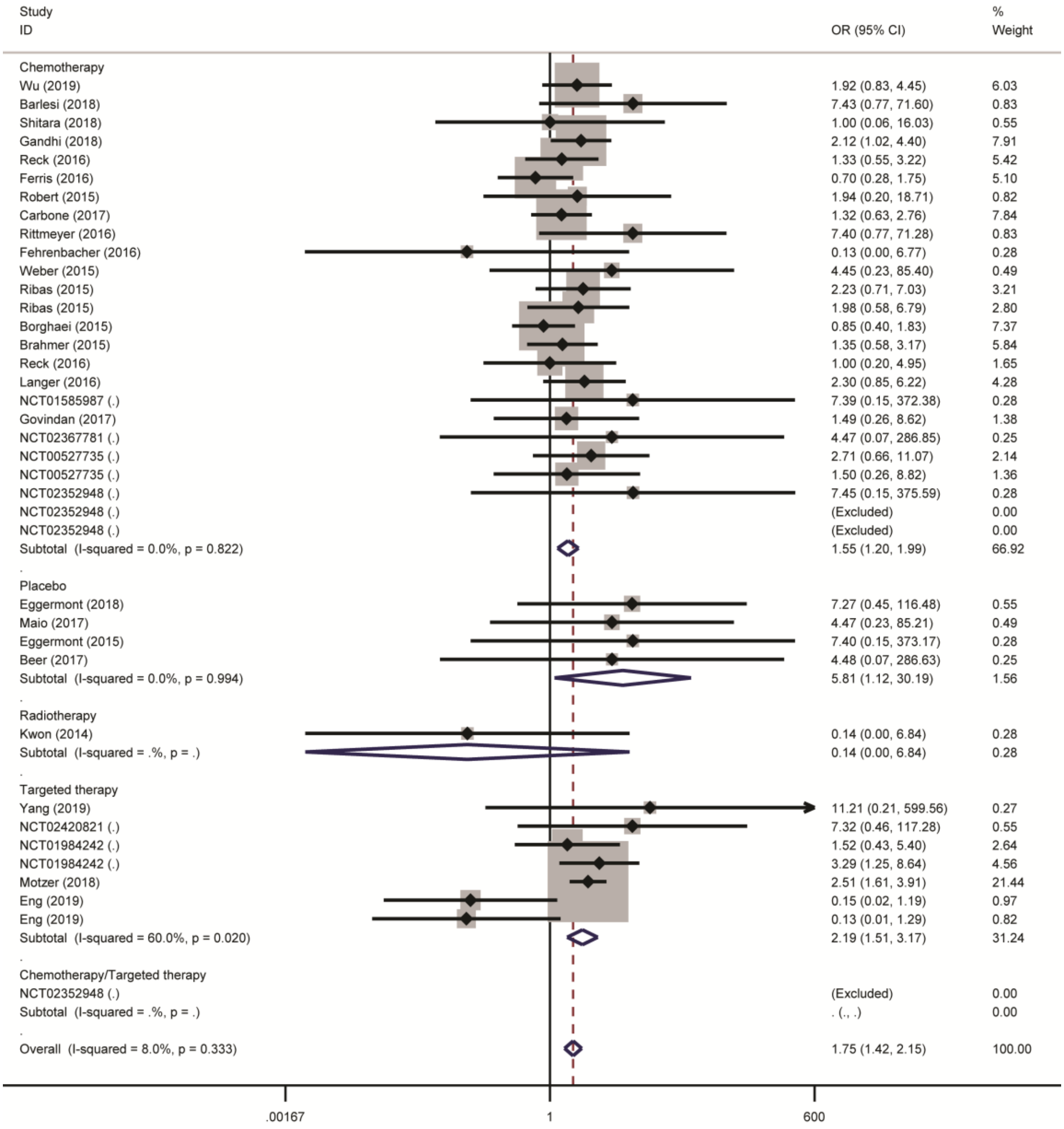

Supplementary Figure S6. Risk of serious-grade hyperglycemia following the use of ICIs versus control treatment, after excluding everolimus-based control group.

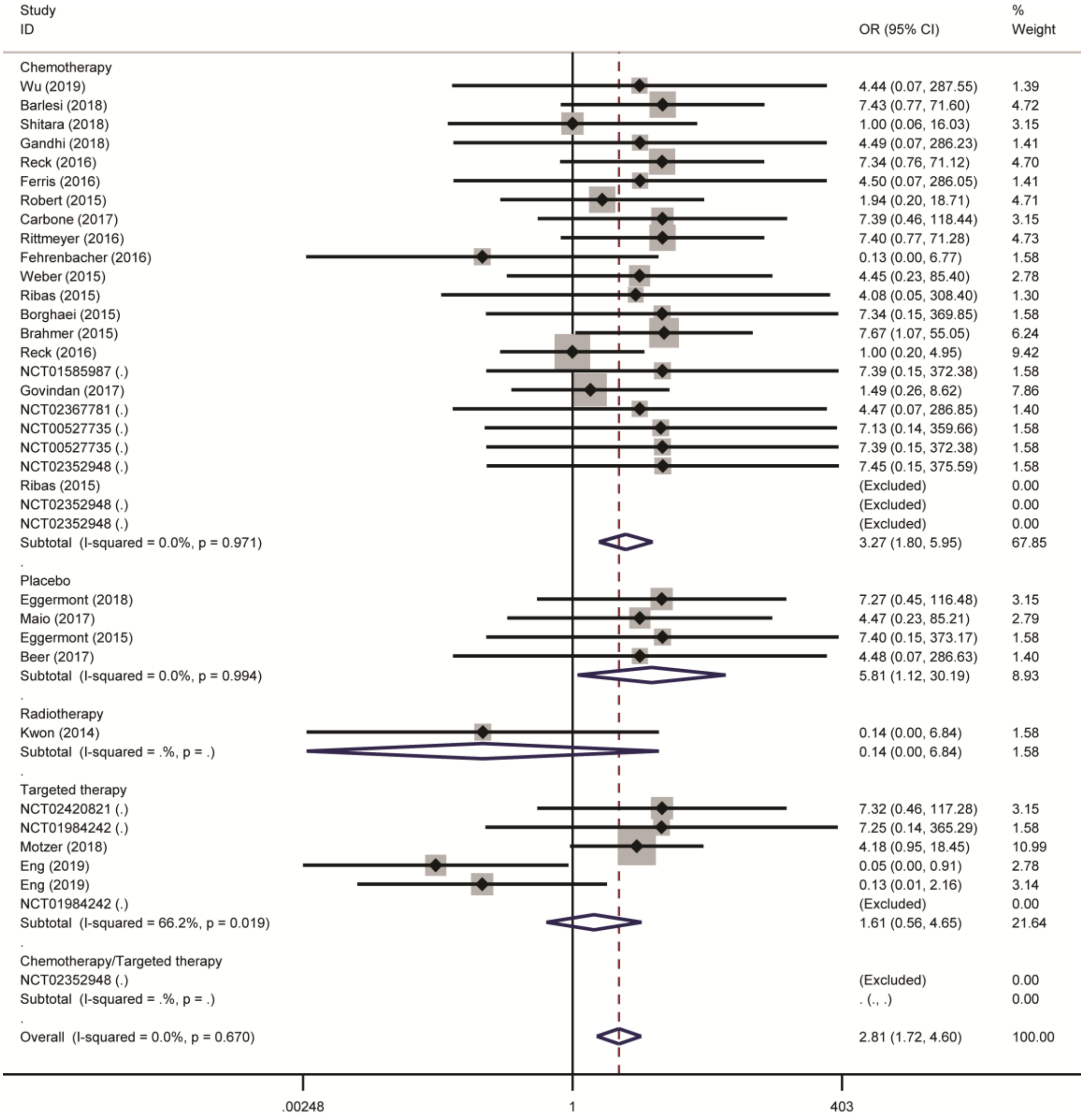

Supplementary Figure S7. Risk of diabetes mellitus following the use of ICIs versus control treatment, stratified by the mode of treatment (monotherapy v add-on therapy).

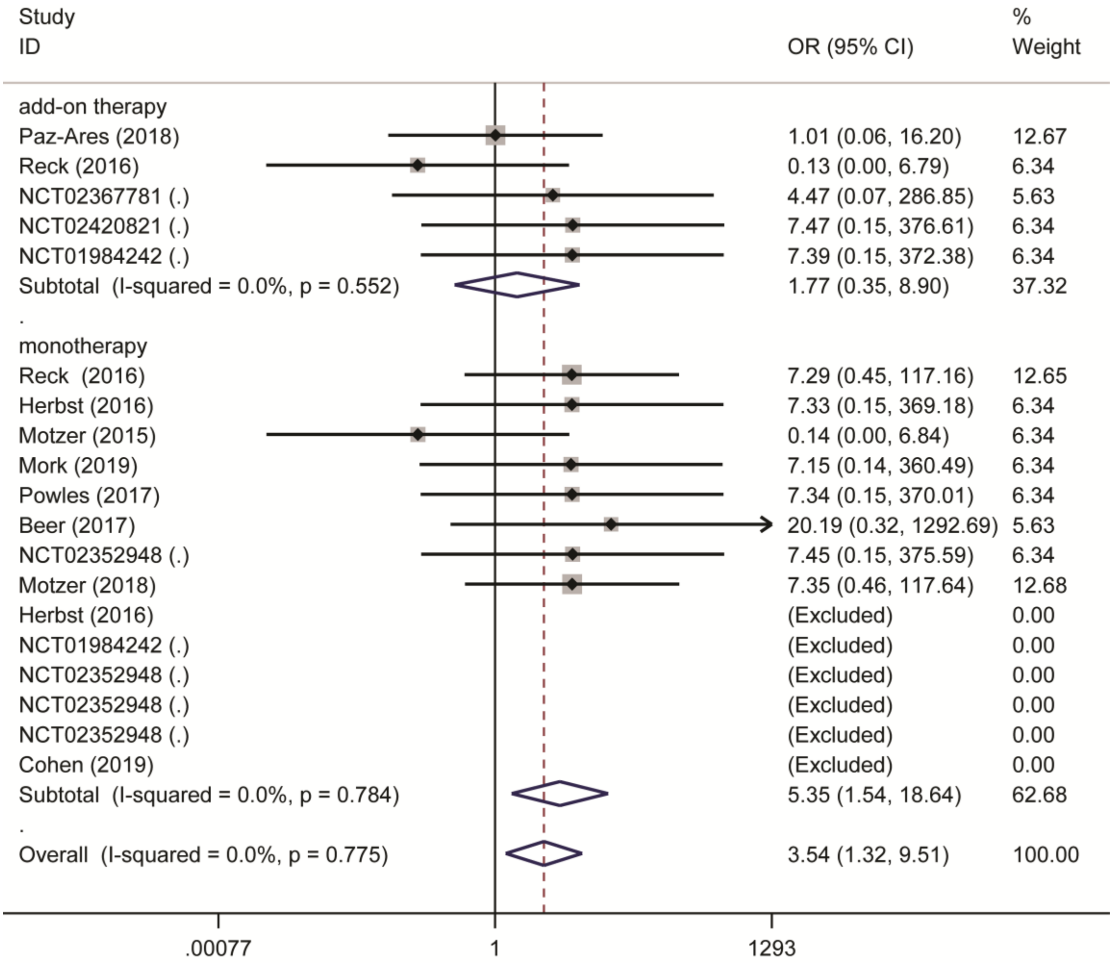

Supplementary Figure S8. Risk of serious-grade hyperglycemia following the use of ICIs versus control treatment, stratified by the mode of treatment (monotherapy v add-on therapy).

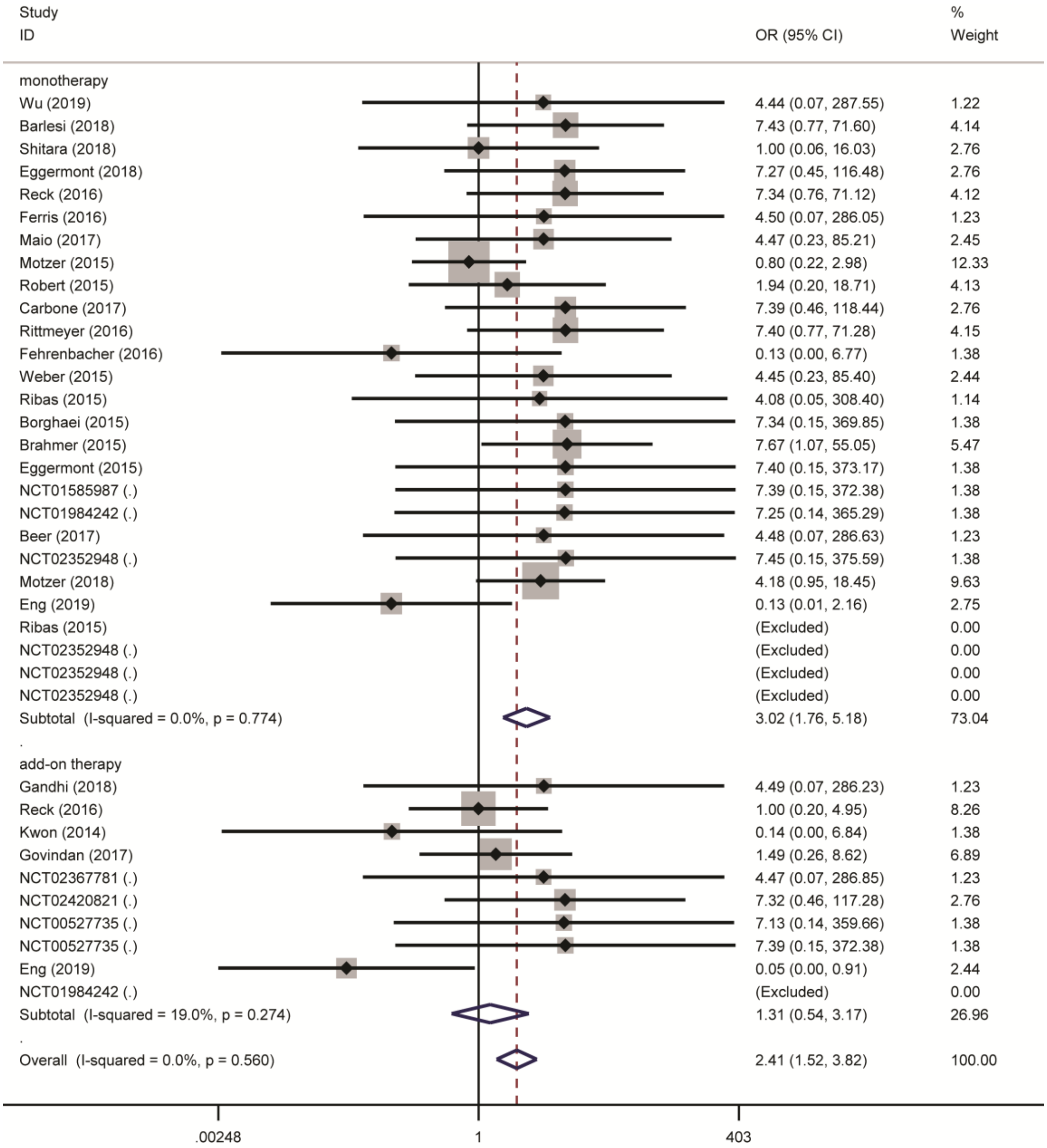

Supplementary Figure S9. Risk of type 2 diabetes following the use of ICIs versus control treatment, stratified by the mode of treatment (monotherapy v add-on therapy).

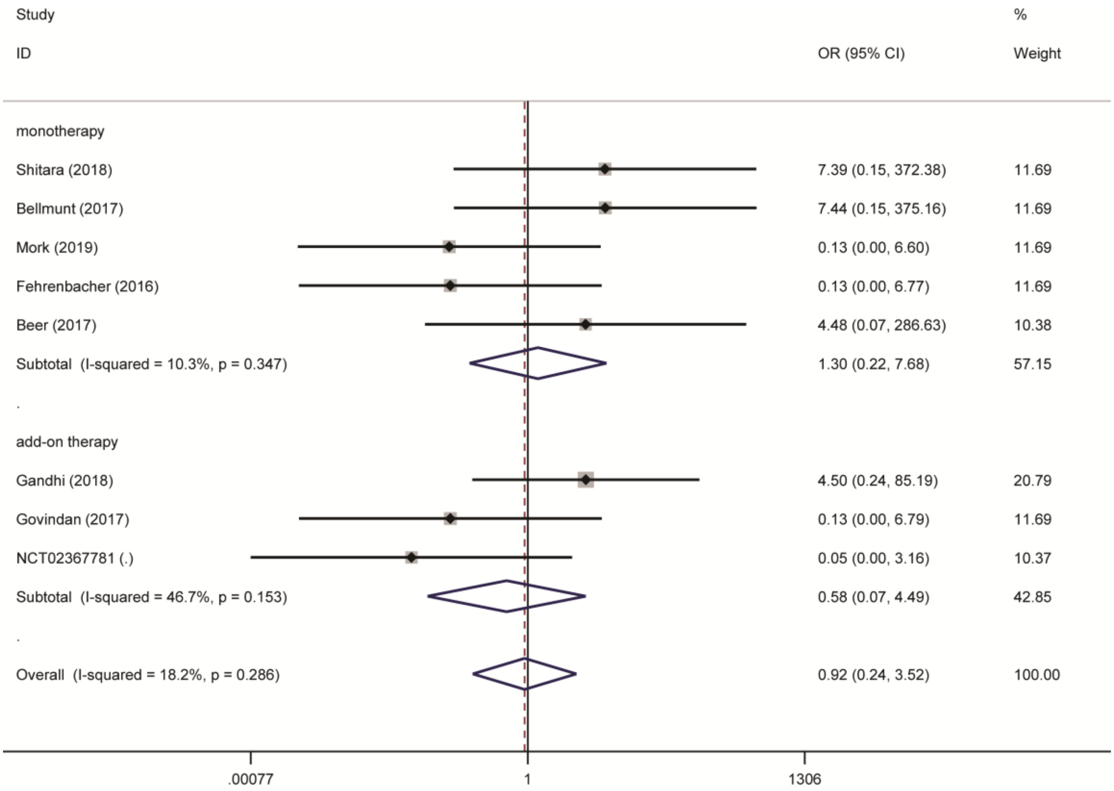

Supplementary Figure S10. Risk of all-grade type 1 diabetes following the use of ICIs versus control treatment, stratified by the mode of treatment (monotherapy v add-on therapy).

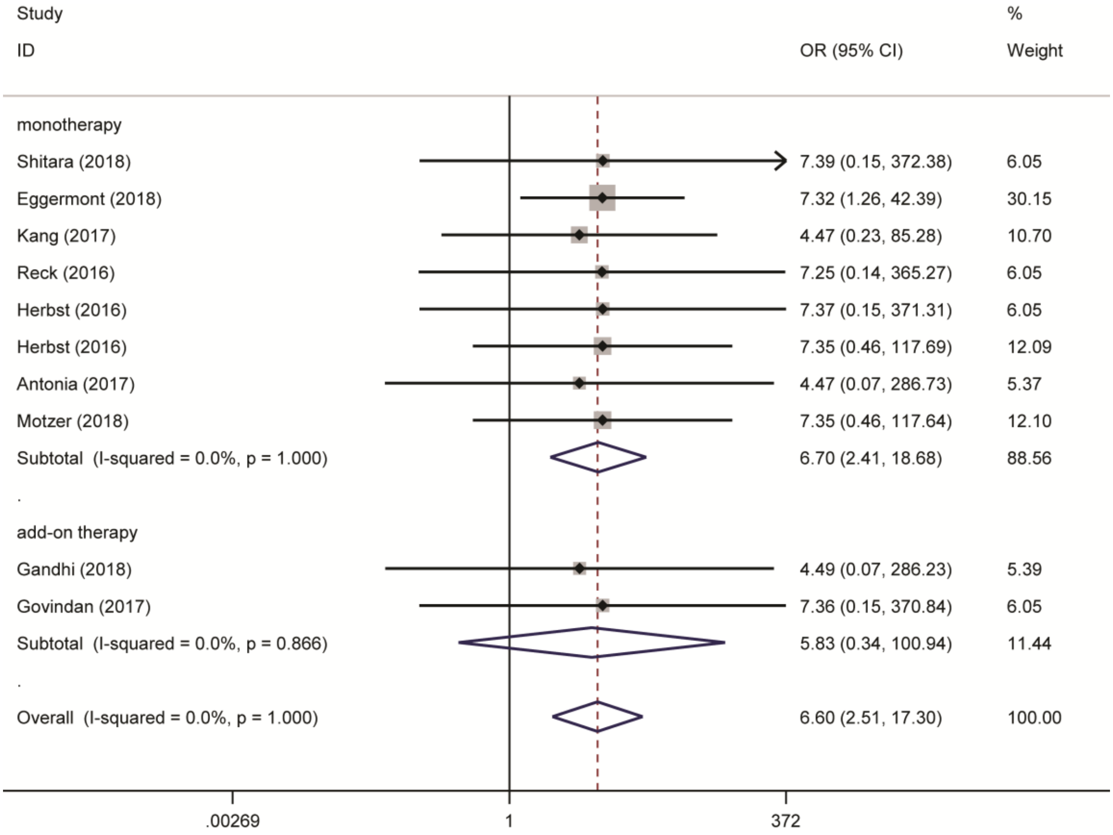

Supplementary Figure S11. Risk of serious type 1 diabetes following the use of ICIs versus control treatment, stratified by the mode of treatment (monotherapy v add-on therapy).

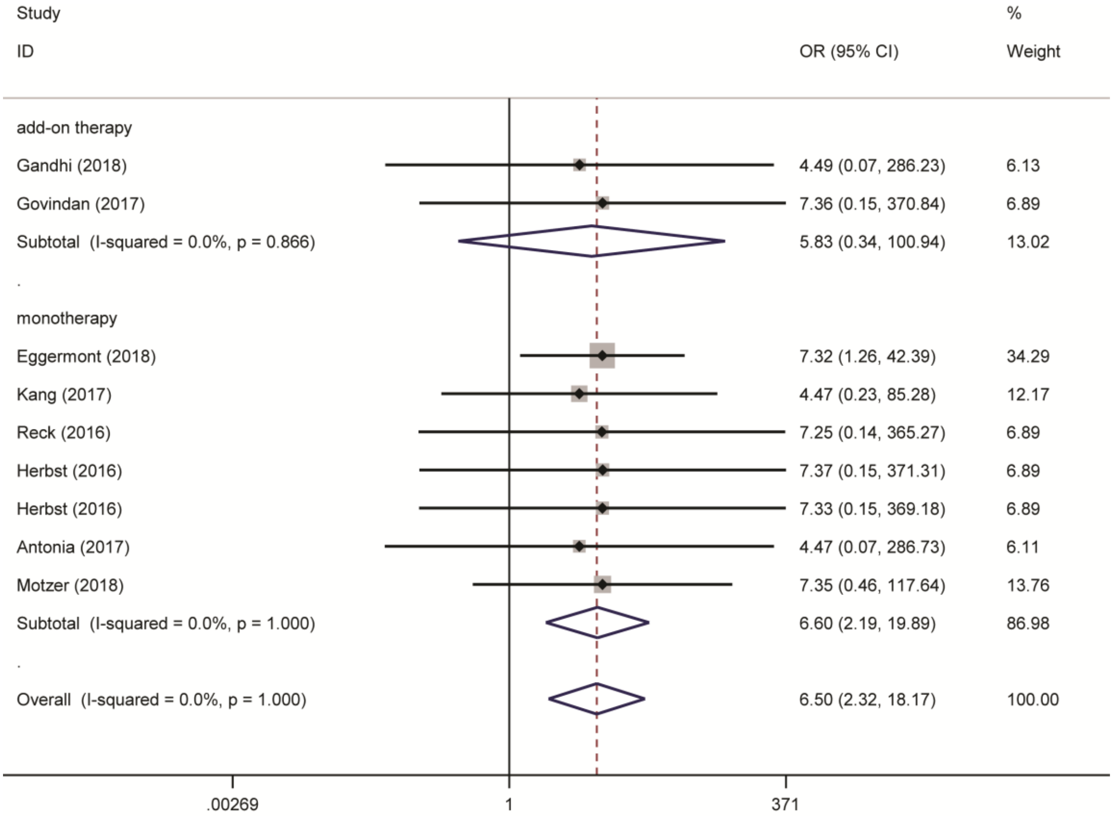

Supplementary Figure S12. Risk of diabetes mellitus following the use of ICIs versus control treatment, stratified by the type of ICIs. (anti-PD-1 v anti-PD-L1 v anti-CTLA-4 v ICI combination)

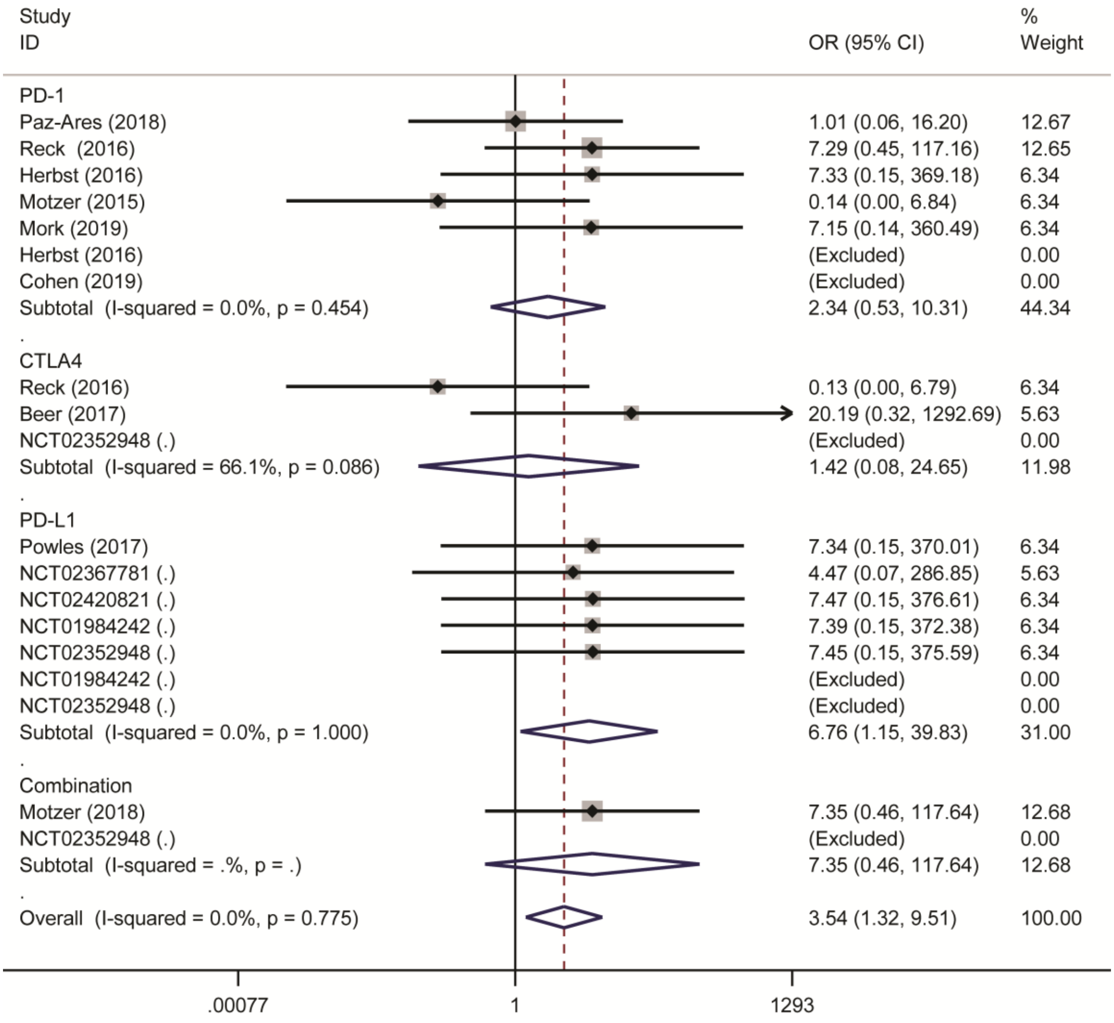

Supplementary Figure S13. Risk of all-grade hyperglycemia following the use of ICIs versus control treatment, stratified by the type of ICIs. (anti-PD-1 v anti-PD-L1 v anti-CTLA-4 v ICI combination)

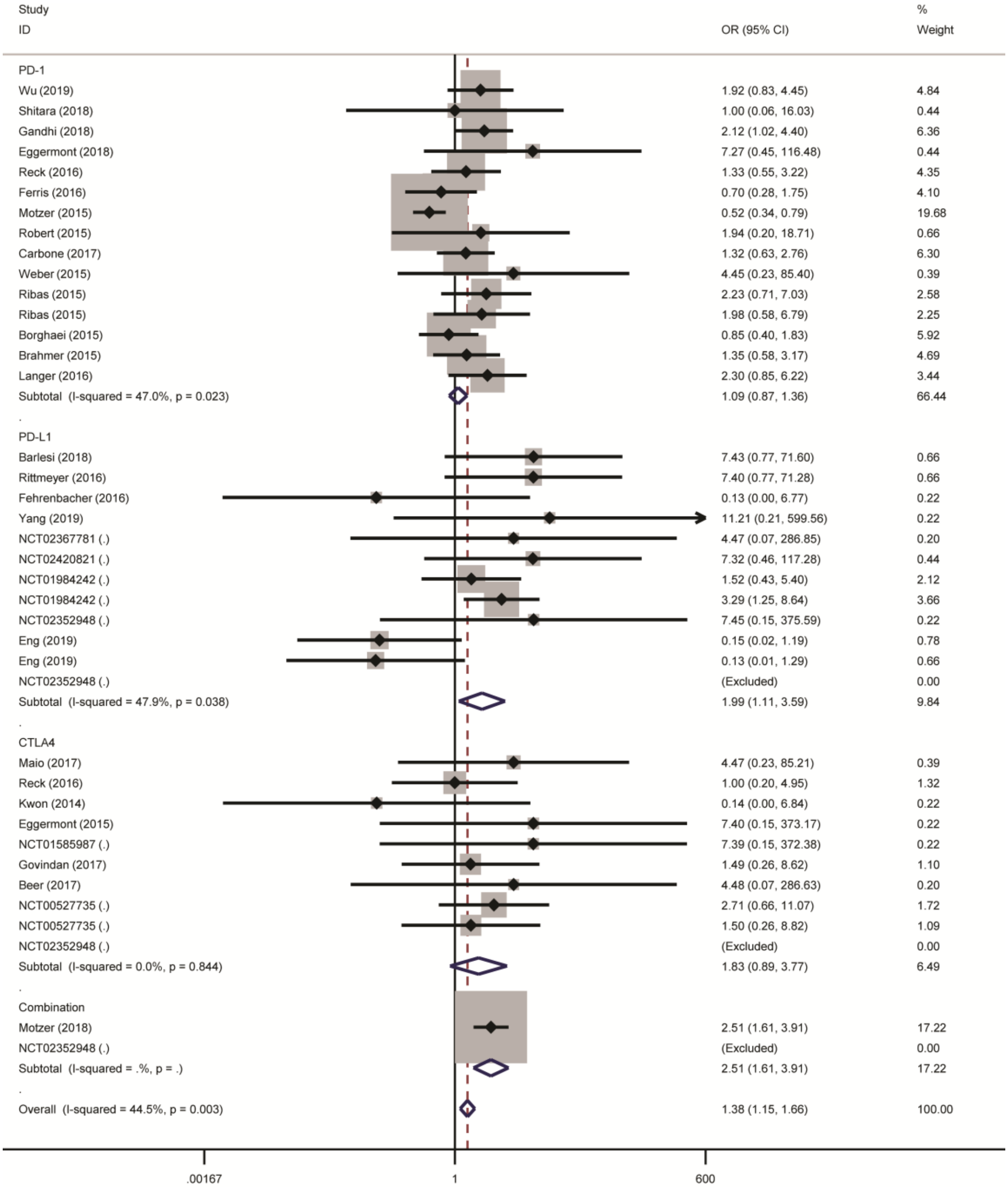

Supplementary Figure S14. Risk of serious-grade hyperglycemia following the use of ICIs versus control treatment, stratified by the type of ICIs. (anti-PD-1 v anti-PD-L1 v anti-CTLA-4 v ICI combination)

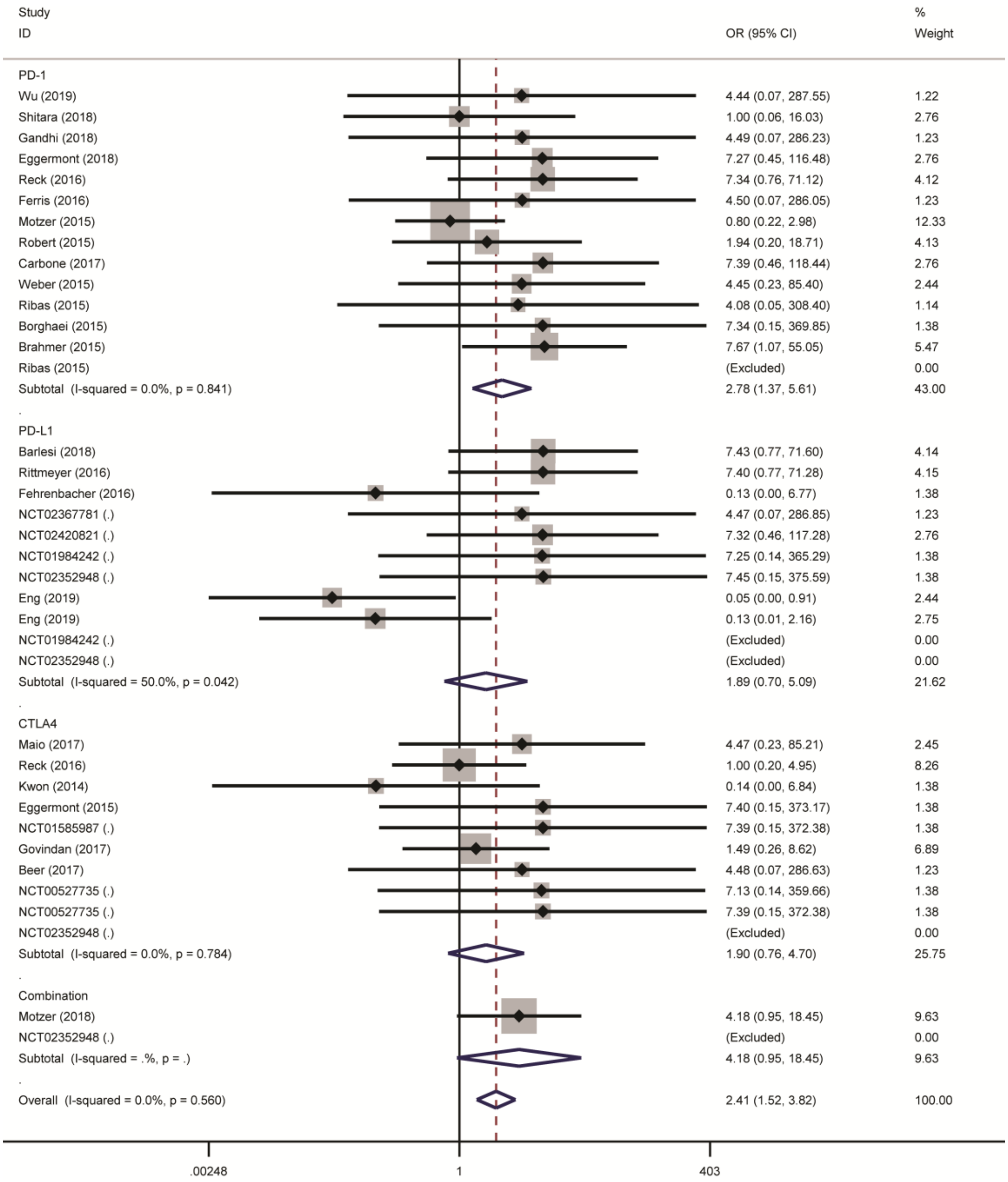

Supplementary Figure S15. Risk of type 2 diabetes following the use of ICIs versus control treatment, stratified by the type of ICIs. (anti-PD-1 v anti-PD-L1 v anti-CTLA-4 v ICI combination)

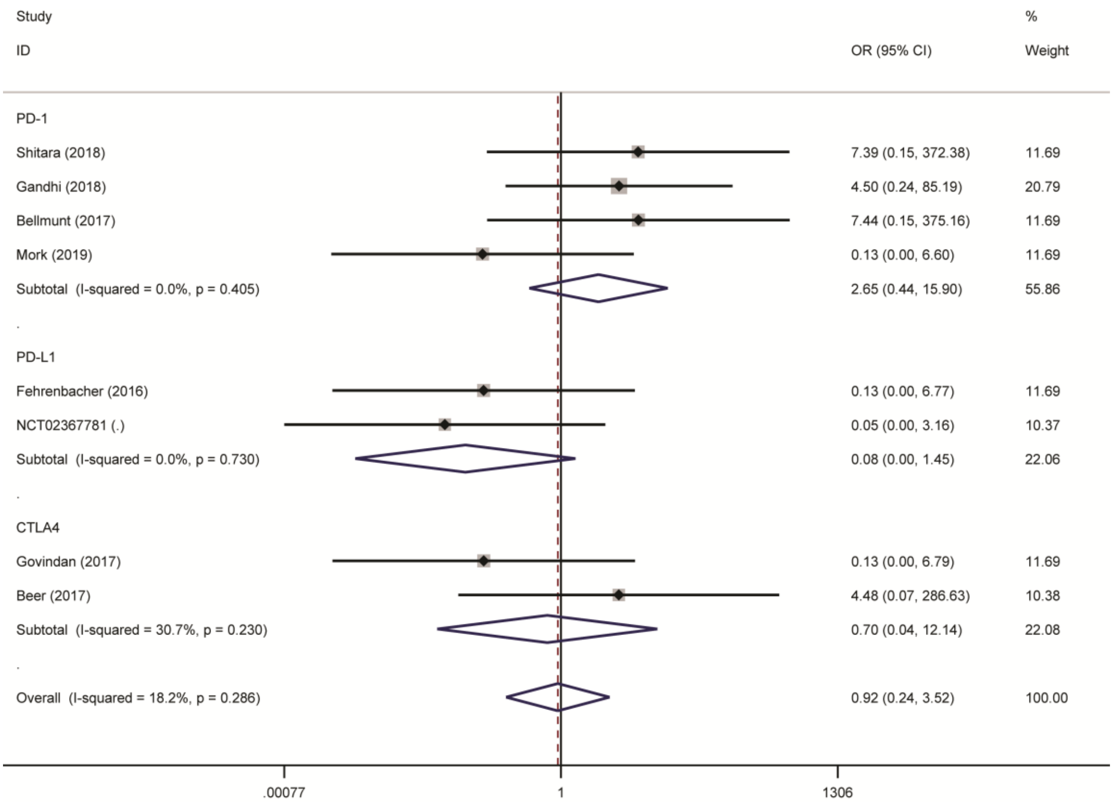

Supplementary Figure S16. Risk of all-grade type 1 diabetes following the use of ICIs versus control treatment, stratified by the type of ICIs. (anti-PD-1 v anti-PD-L1 v anti-CTLA-4 v ICI combination)

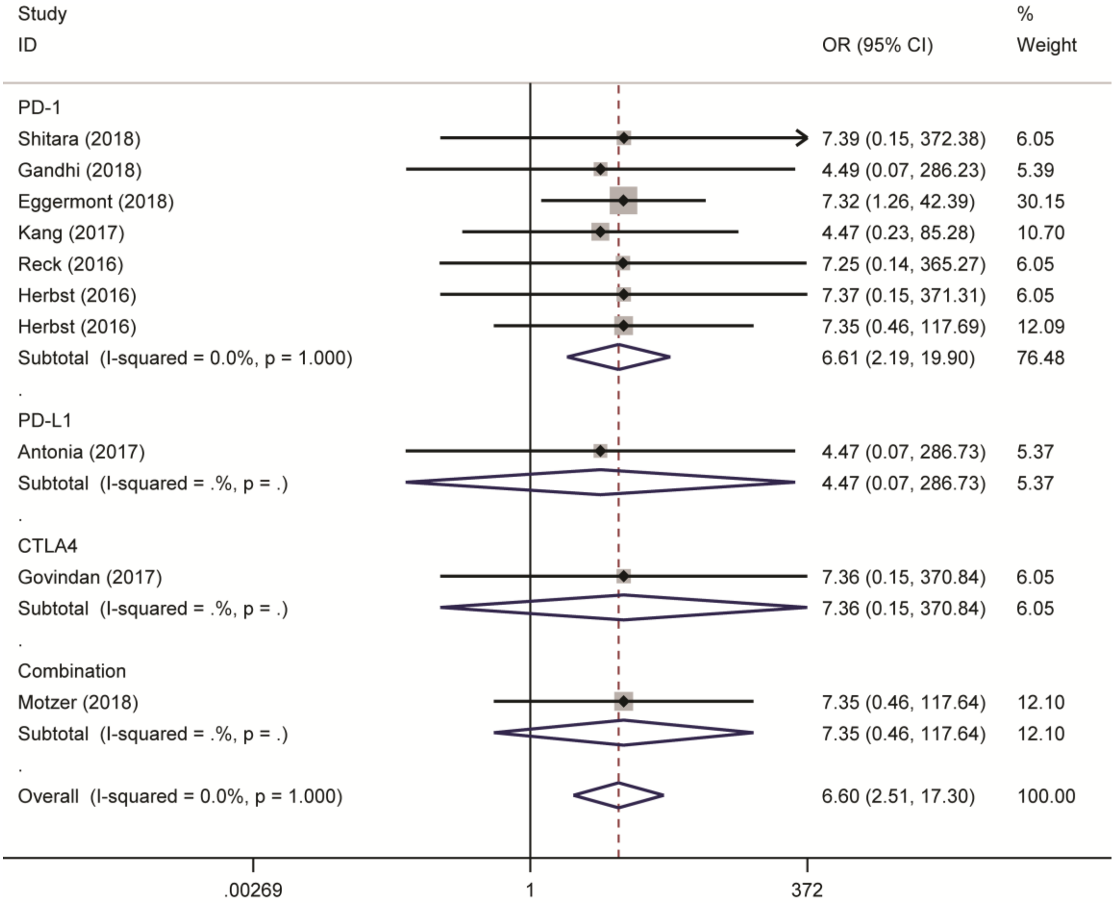

Supplementary Figure S17. Risk of serious-grade type 1 diabetes following the use of ICIs versus control treatment, stratified by the type of ICIs. (anti-PD-1 v anti-PD-L1 v anti-CTLA-4 v ICI combination)

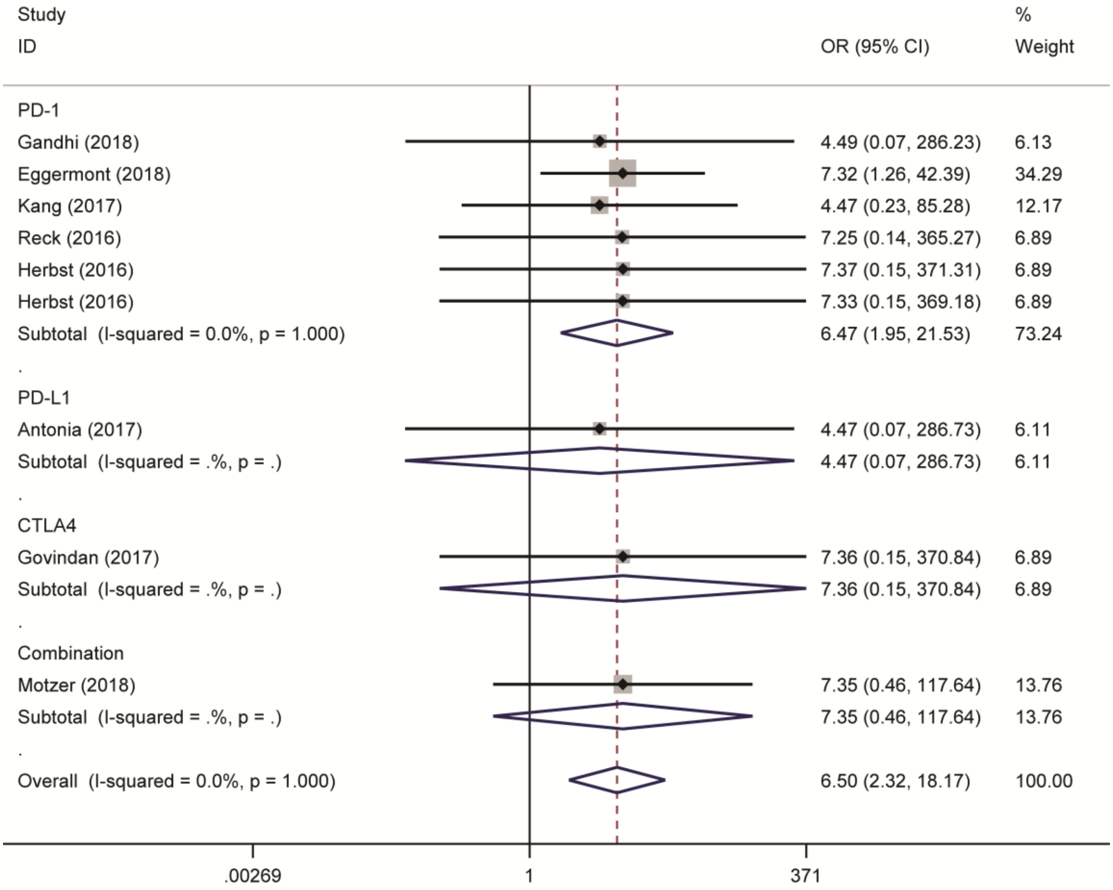

Supplementary Figure S18. Funnel plot of meta-analysis for diabetes mellitus

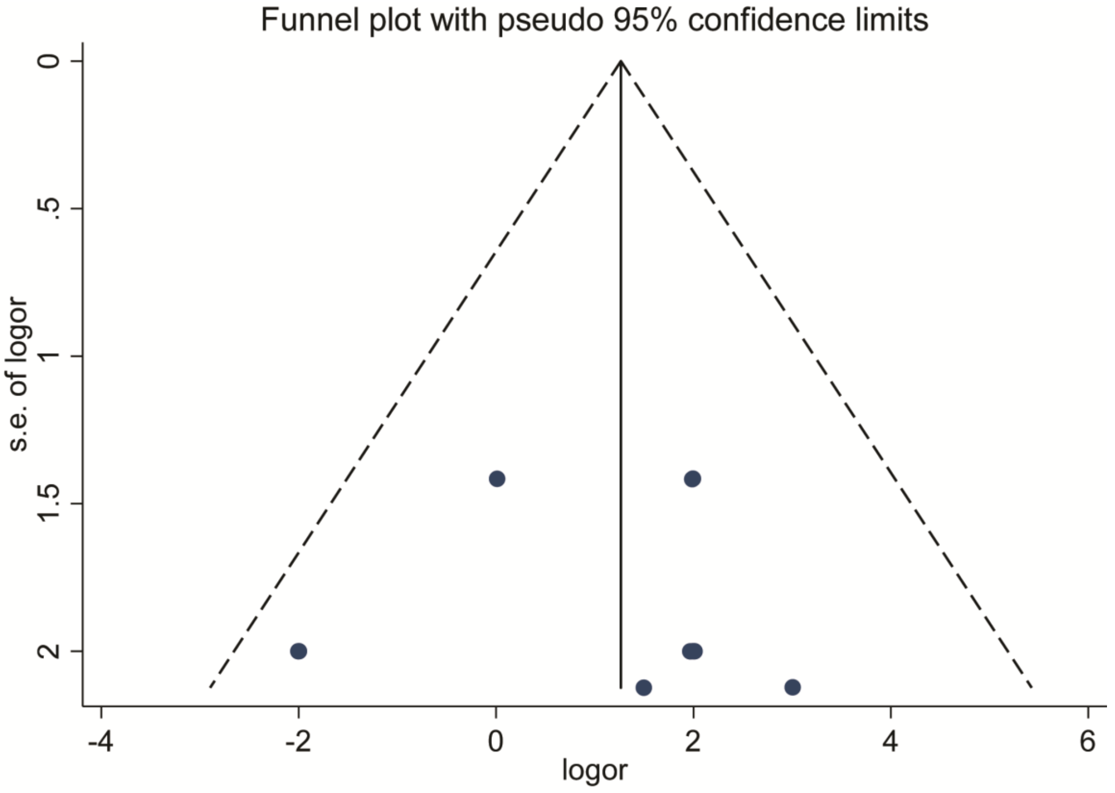

Supplementary Figure S19. Funnel plot of meta-analysis for all-grade hyperglycemia

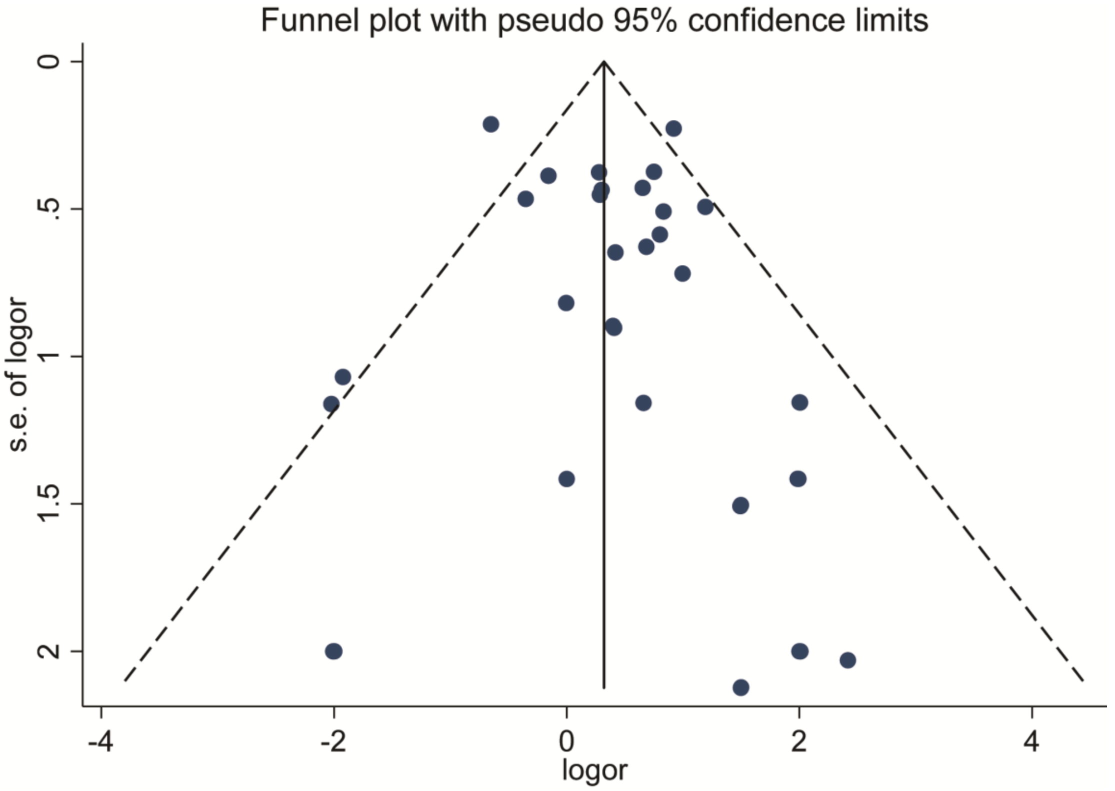

Supplementary Figure S20. Funnel plot of meta-analysis for serious-grade hyperglycemia

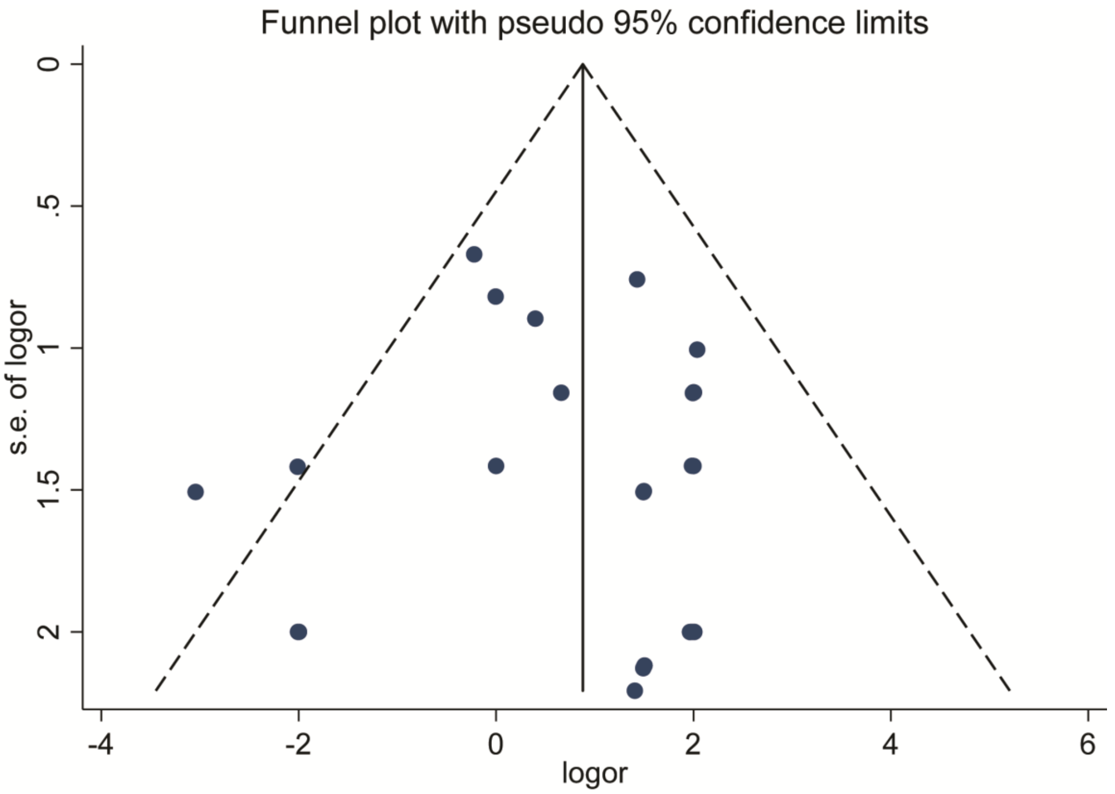

Supplementary Figure S21. Funnel plot of meta-analysis for type 2 diabetes

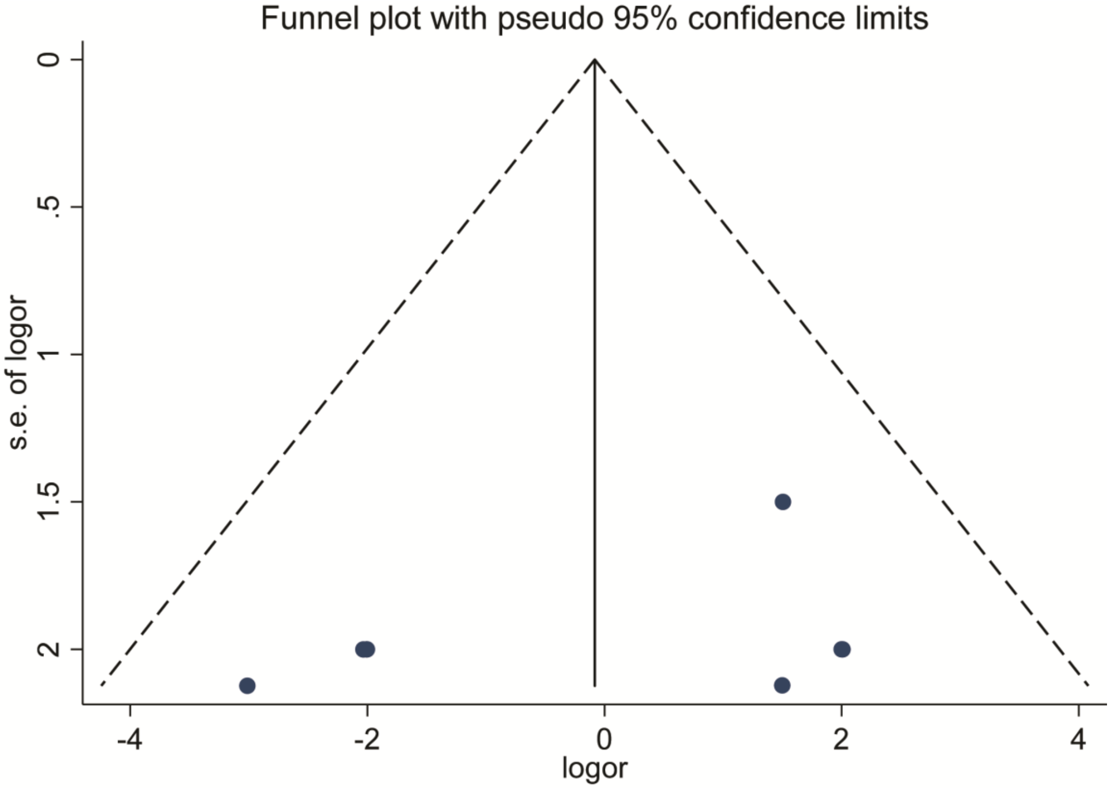

Supplementary Figure S22. Funnel plot of meta-analysis for all-grade type 1 diabetes

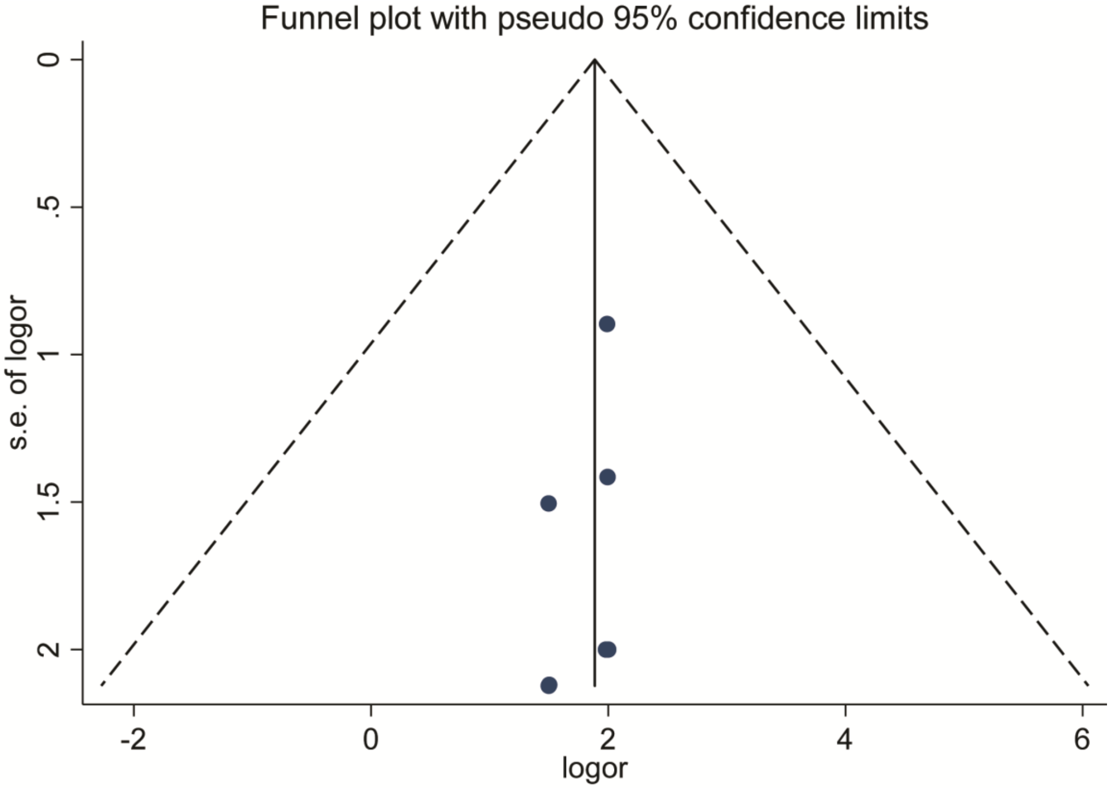

Supplementary Figure S23. Funnel plot of meta-analysis for serious-grade type 1 diabetes

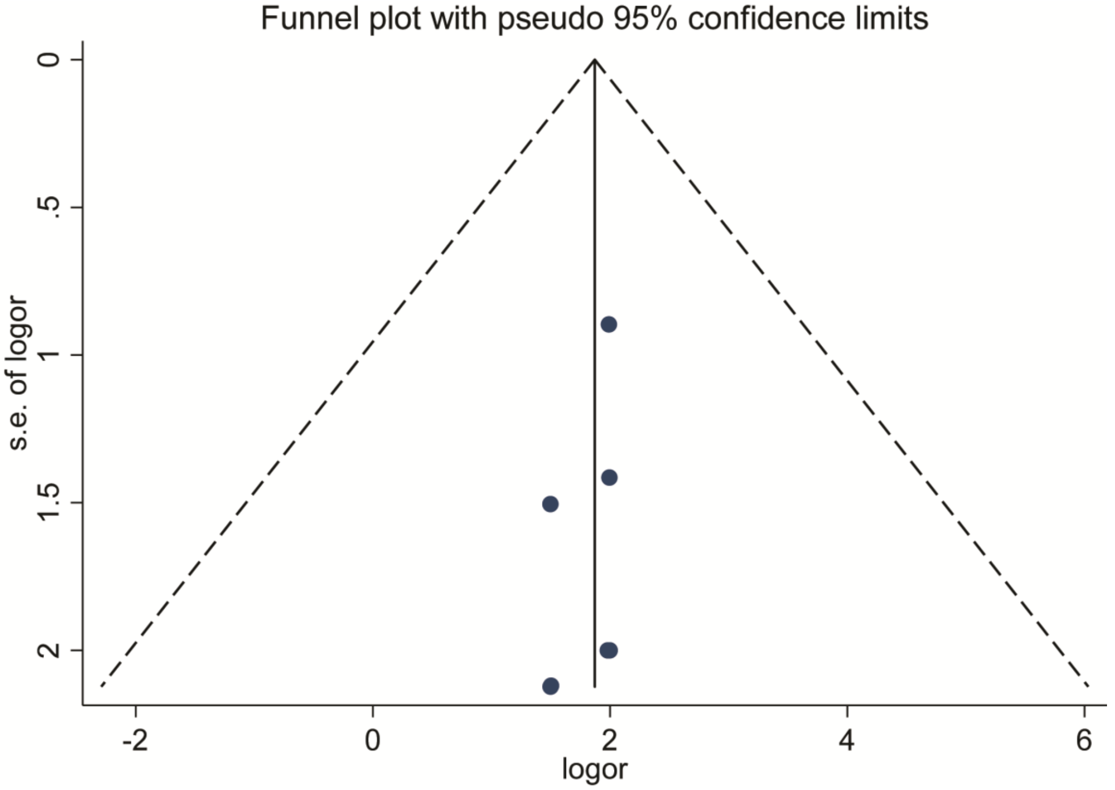

Reference:

Antonia, S.J., Villegas, A., Daniel, D., Vicente, D., Murakami, S., Hui, R., et al. (2017). Durvalumab after Chemoradiotherapy in Stage III Non-Small-Cell Lung Cancer. *N Engl J Med* 377(20)**,** 1919-1929. doi: 10.1056/NEJMoa1709937.

AstraZeneca (2015). "A Global Study to Assess the Effects of MEDI4736 (Durvalumab), Given as Monotherapy or in Combination With Tremelimumab Determined by PD-L1 Expression Versus Standard of Care in Patients With Locally Advanced or Metastatic Non Small Cell Lung Cancer". https://ClinicalTrials.gov/show/NCT02352948).

Barlesi, F., Vansteenkiste, J., Spigel, D., Ishii, H., Garassino, M., de Marinis, F., et al. (2018). Avelumab versus docetaxel in patients with platinum-treated advanced non-small-cell lung cancer (JAVELIN Lung 200): an open-label, randomised, phase 3 study. *Lancet Oncol* 19(11)**,** 1468-1479. doi: 10.1016/S1470-2045(18)30673-9.

Beer, T.M., Kwon, E.D., Drake, C.G., Fizazi, K., Logothetis, C., Gravis, G., et al. (2017). Randomized, Double-Blind, Phase III Trial of Ipilimumab Versus Placebo in Asymptomatic or Minimally Symptomatic Patients With Metastatic Chemotherapy-Naive Castration-Resistant Prostate Cancer. *J Clin Oncol* 35(1)**,** 40-47. doi: 10.1200/JCO.2016.69.1584.

Borghaei, H., Paz-Ares, L., Horn, L., Spigel, D.R., Steins, M., Ready, N.E., et al. (2015). Nivolumab versus Docetaxel in Advanced Nonsquamous Non-Small-Cell Lung Cancer. *N Engl J Med* 373(17)**,** 1627-1639. doi: 10.1056/NEJMoa1507643.

Brahmer, J., Reckamp, K.L., Baas, P., Crino, L., Eberhardt, W.E., Poddubskaya, E., et al. (2015). Nivolumab versus Docetaxel in Advanced Squamous-Cell Non-Small-Cell Lung Cancer. *N Engl J Med* 373(2)**,** 123-135. doi: 10.1056/NEJMoa1504627.

Carbone, D.P., Reck, M., Paz-Ares, L., Creelan, B., Horn, L., Steins, M., et al. (2017). First-Line Nivolumab in Stage IV or Recurrent Non-Small-Cell Lung Cancer. *N Engl J Med* 376(25)**,** 2415-2426. doi: 10.1056/NEJMoa1613493.

Chih-Hsin Yang, J., Shepherd, F.A., Kim, D.W., Lee, G.W., Lee, J.S., Chang, G.C., et al. (2019). Osimertinib Plus Durvalumab versus Osimertinib Monotherapy in EGFR T790M-Positive NSCLC following Previous EGFR TKI Therapy: CAURAL Brief Report. *J Thorac Oncol* 14(5)**,** 933-939. doi: 10.1016/j.jtho.2019.02.001.

Cohen, E.E.W., Soulieres, D., Le Tourneau, C., Dinis, J., Licitra, L., Ahn, M.J., et al. (2019). Pembrolizumab versus methotrexate, docetaxel, or cetuximab for recurrent or metastatic head-and-neck squamous cell carcinoma (KEYNOTE-040): a randomised, open-label, phase 3 study. *Lancet* 393(10167)**,** 156-167. doi: 10.1016/S0140-6736(18)31999-8.

Eggermont, A.M., Chiarion-Sileni, V., Grob, J.J., Dummer, R., Wolchok, J.D., Schmidt, H., et al. (2015). Adjuvant ipilimumab versus placebo after complete resection of high-risk stage III melanoma (EORTC 18071): a randomised, double-blind, phase 3 trial. *Lancet Oncol* 16(5)**,** 522-530. doi: 10.1016/S1470-2045(15)70122-1.

Eggermont, A.M.M., Blank, C.U., Mandala, M., Long, G.V., Atkinson, V., Dalle, S., et al. (2018). Adjuvant Pembrolizumab versus Placebo in Resected Stage III Melanoma. *N Engl J Med* 378(19)**,** 1789-1801. doi: 10.1056/NEJMoa1802357.

Eng, C., Kim, T.W., Bendell, J., Argiles, G., Tebbutt, N.C., Di Bartolomeo, M., et al. (2019). Atezolizumab with or without cobimetinib versus regorafenib in previously treated metastatic colorectal cancer (IMblaze370): a multicentre, open-label, phase 3, randomised, controlled trial. *Lancet Oncol*. doi: 10.1016/S1470-2045(19)30027-0.

Fehrenbacher, L., Spira, A., Ballinger, M., Kowanetz, M., Vansteenkiste, J., Mazieres, J., et al. (2016). Atezolizumab versus docetaxel for patients with previously treated non-small-cell lung cancer (POPLAR): a multicentre, open-label, phase 2 randomised controlled trial. *Lancet* 387(10030), 1837-1846. doi: 10.1016/S0140-6736(16)00587-0.

Ferris, R.L., Blumenschein, G., Jr., Fayette, J., Guigay, J., Colevas, A.D., Licitra, L., et al. (2016). Nivolumab for Recurrent Squamous-Cell Carcinoma of the Head and Neck. *N Engl J Med* 375(19), 1856-1867. doi: 10.1056/NEJMoa1602252.

Gandhi, L., Rodriguez-Abreu, D., Gadgeel, S., Esteban, E., Felip, E., De Angelis, F., et al. (2018). Pembrolizumab plus Chemotherapy in Metastatic Non-Small-Cell Lung Cancer. *N Engl J Med* 378(22), 2078-2092. doi: 10.1056/NEJMoa1801005.

Govindan, R., Szczesna, A., Ahn, M.J., Schneider, C.P., Gonzalez Mella, P.F., Barlesi, F., et al. (2017). Phase III Trial of Ipilimumab Combined With Paclitaxel and Carboplatin in Advanced Squamous Non-Small-Cell Lung Cancer. *J Clin Oncol* 35(30), 3449-3457. doi: 10.1200/JCO.2016.71.7629.

Herbst, R.S., Baas, P., Kim, D.W., Felip, E., Perez-Gracia, J.L., Han, J.Y., et al. (2016). Pembrolizumab versus docetaxel for previously treated, PD-L1-positive, advanced non-small-cell lung cancer (KEYNOTE-010): a randomised controlled trial. *Lancet* 387(10027), 1540-1550. doi: 10.1016/S0140-6736(15)01281-7.

Kang, Y.K., Boku, N., Satoh, T., Ryu, M.H., Chao, Y., Kato, K., et al. (2017). Nivolumab in patients with advanced gastric or gastro-oesophageal junction cancer refractory to, or intolerant of, at least two previous chemotherapy regimens (ONO-4538-12, ATTRACTION-2): a randomised, double-blind, placebo-controlled, phase 3 trial. *Lancet* 390(10111), 2461-2471. doi: 10.1016/S0140-6736(17)31827-5.

Kwon, E.D., Drake, C.G., Scher, H.I., Fizazi, K., Bossi, A., van den Eertwegh, A.J., et al. (2014). Ipilimumab versus placebo after radiotherapy in patients with metastatic castration-resistant prostate cancer that had progressed after docetaxel chemotherapy (CA184-043): a multicentre, randomised, double-blind, phase 3 trial. *Lancet Oncol* 15(7), 700-712. doi: 10.1016/S1470-2045(14)70189-5.

Langer, C.J., Gadgeel, S.M., Borghaei, H., Papadimitrakopoulou, V.A., Patnaik, A., Powell, S.F., et al. (2016). Carboplatin and pemetrexed with or without pembrolizumab for advanced, non-squamous non-small-cell lung cancer: a randomised, phase 2 cohort of the open-label KEYNOTE-021 study. *Lancet Oncol* 17(11), 1497-1508. doi: 10.1016/S1470-2045(16)30498-3.

Llombart-Cussac, A., Cortes, J., Pare, L., Galvan, P., Bermejo, B., Martinez, N., et al. (2017). HER2-enriched subtype as a predictor of pathological complete response following trastuzumab and lapatinib without chemotherapy in early-stage HER2-positive breast cancer (PAMELA): an open-label, single-group, multicentre, phase 2 trial. *Lancet Oncol* 18(4), 545-554. doi: 10.1016/S1470-2045(17)30021-9.

Mok, T.S.K., Wu, Y.L., Kudaba, I., Kowalski, D.M., Cho, B.C., Turna, H.Z., et al. (2019). Pembrolizumab versus chemotherapy for previously untreated, PD-L1-expressing, locally advanced or metastatic non-small-cell lung cancer (KEYNOTE-042): a randomised, open-label, controlled, phase 3 trial. *Lancet* 393(10183), 1819-1830. doi: 10.1016/S0140-6736(18)32409-7.

Motzer, R.J., Escudier, B., McDermott, D.F., George, S., Hammers, H.J., Srinivas, S., et al. (2015). Nivolumab versus Everolimus in Advanced Renal-Cell Carcinoma. *N Engl J Med* 373(19), 1803-1813. doi: 10.1056/NEJMoa1510665.

Motzer, R.J., Tannir, N.M., McDermott, D.F., Aren Frontera, O., Melichar, B., Choueiri, T.K., et al. (2018). Nivolumab plus Ipilimumab versus Sunitinib in Advanced Renal-Cell Carcinoma. *N Engl J Med* 378(14), 1277-1290. doi: 10.1056/NEJMoa1712126.

Paz-Ares, L., Luft, A., Vicente, D., Tafreshi, A., Gumus, M., Mazieres, J., et al. (2018). Pembrolizumab plus Chemotherapy for Squamous Non-Small-Cell Lung Cancer. *N Engl J Med* 379(21), 2040-2051. doi: 10.1056/NEJMoa1810865.

Powles, T., Duran, I., van der Heijden, M.S., Loriot, Y., Vogelzang, N.J., De Giorgi, U., et al. (2018). Atezolizumab versus chemotherapy in patients with platinum-treated locally advanced or metastatic urothelial carcinoma (IMvigor211): a multicentre, open-label, phase 3 randomised controlled trial. *Lancet* 391(10122), 748-757. doi: 10.1016/S0140-6736(17)33297-X.

Reck, M., Bondarenko, I., Luft, A., Serwatowski, P., Barlesi, F., Chacko, R., et al. (2013). Ipilimumab in combination with paclitaxel and carboplatin as first-line therapy in extensive-disease-small-cell lung cancer: results from a randomized, double-blind, multicenter phase 2 trial. *Ann Oncol* 24(1), 75-83. doi: 10.1093/annonc/mds213.

Reck, M., Luft, A., Szczesna, A., Havel, L., Kim, S.W., Akerley, W., et al. (2016a). Phase III Randomized Trial of Ipilimumab Plus Etoposide and Platinum Versus Placebo Plus Etoposide and Platinum in Extensive-Stage Small-Cell Lung Cancer. *J Clin Oncol* 34(31), 3740-3748. doi: 10.1200/JCO.2016.67.6601.

Reck, M., Rodriguez-Abreu, D., Robinson, A.G., Hui, R., Csoszi, T., Fulop, A., et al. (2016b). Pembrolizumab versus Chemotherapy for PD-L1-Positive Non-Small-Cell Lung Cancer. *N Engl J Med* 375(19), 1823-1833. doi: 10.1056/NEJMoa1606774.

Ribas, A., Puzanov, I., Dummer, R., Schadendorf, D., Hamid, O., Robert, C., et al. (2015). Pembrolizumab versus investigator-choice chemotherapy for ipilimumab-refractory melanoma (KEYNOTE-002): a randomised, controlled, phase 2 trial. *Lancet Oncol* 16(8), 908-918. doi: 10.1016/S1470-2045(15)00083-2.

Rittmeyer, A., Barlesi, F., Waterkamp, D., Park, K., Ciardiello, F., von Pawel, J., et al. (2017). Atezolizumab versus docetaxel in patients with previously treated non-small-cell lung cancer (OAK): a phase 3, open-label, multicentre randomised controlled trial. *Lancet* 389(10066), 255-265. doi: 10.1016/S0140-6736(16)32517-X.

Robert, C., Long, G.V., Brady, B., Dutriaux, C., Maio, M., Mortier, L., et al. (2015). Nivolumab in previously untreated melanoma without BRAF mutation. *N Engl J Med* 372(4), 320-330. doi: 10.1056/NEJMoa1412082.

Roche, H.-L. (2014). "A Study of Atezolizumab (an Engineered Anti-Programmed Death-Ligand 1 PD-L1 Antibody) as Monotherapy or in Combination With Bevacizumab (Avastin®) Compared to Sunitinib (Sutent®) in Participants With Untreated Advanced Renal Cell Carcinoma". https://ClinicalTrials.gov/show/NCT01984242).

Roche, H.-L. (2015a). "A Study of Atezolizumab in Combination With Bevacizumab Versus Sunitinib in Participants With Untreated Advanced Renal Cell Carcinoma (RCC)". https://ClinicalTrials.gov/show/NCT02420821).

Roche, H.-L. (2015b). "A Study of Atezolizumab in Combination With Carboplatin Plus (+) Nab-Paclitaxel Compared With Carboplatin+Nab-Paclitaxel in Participants With Stage IV Non-Squamous Non-Small Cell Lung Cancer (NSCLC)". https://ClinicalTrials.gov/show/NCT02367781).

Rogers, J.G., Pagani, F.D., Tatooles, A.J., Bhat, G., Slaughter, M.S., Birks, E.J., et al. (2017).

Intrapericardial Left Ventricular Assist Device for Advanced Heart Failure. *N Engl J Med* 376(5), 451-460. doi: 10.1056/NEJMoa1602954.

Shitara, K., Ozguroglu, M., Bang, Y.J., Di Bartolomeo, M., Mandala, M., Ryu, M.H., et al. (2018). Pembrolizumab versus paclitaxel for previously treated, advanced gastric or gastro-oesophageal junction cancer (KEYNOTE-061): a randomised, open-label, controlled, phase 3 trial. *Lancet* 392(10142), 123-133. doi: 10.1016/S0140-6736(18)31257-1.

Squibb, B.-M. (2012). "An Efficacy Study in Gastric and Gastroesophageal Junction Cancer Comparing Ipilimumab Versus Standard of Care Immediately Following First Line Chemotherapy". https://ClinicalTrials.gov/show/NCT01585987).

Weber, J.S., D'Angelo, S.P., Minor, D., Hodi, F.S., Gutzmer, R., Neyns, B., et al. (2015). Nivolumab versus chemotherapy in patients with advanced melanoma who progressed after anti-CTLA-4 treatment (CheckMate 037): a randomised, controlled, open-label, phase 3 trial. *Lancet Oncol* 16(4), 375-384. doi: 10.1016/S1470-2045(15)70076-8.

Wu, Y.L., Lu, S., Cheng, Y., Zhou, C., Wang, J., Mok, T., et al. (2019). Nivolumab Versus Docetaxel in a Predominantly Chinese Patient Population With Previously Treated Advanced NSCLC: CheckMate 078 Randomized Phase III Clinical Trial. *J Thorac Oncol* 14(5), 867-875. doi: 10.1016/j.jtho.2019.01.006.
